# Anti-EGFR ScFv functionalized exosomes delivering LPCAT1 specific siRNAs for inhibition of lung cancer brain metastases

**DOI:** 10.1186/s12951-024-02414-7

**Published:** 2024-04-08

**Authors:** Jun Jiang, Yuan Lu, Jie Chu, Xiao Zhang, Chao Xu, Shaojie Liu, Zhuo Wan, Jiawei Wang, Lu Zhang, Kui Liu, Zhenhua Liu, Angang Yang, Xinling Ren, Rui Zhang

**Affiliations:** 1 Department of Health Service, Base of Health Service, Air Force Medical University, Xi’an, China; 2 Department of Respiratory and Critical Care Medicine, Zhongda Hospital, Southeast University, Nanjing, 210009 China; 3 State Key Laboratory of Cancer Biology, Department of Biochemistry and Molecular Biology, Air Force Medical University, Xi’an, China; 4 Department of Urology, Xijing Hospital, Air Force Medical University, Xi’an, China; 5 Department of Hematology, Tangdu Hospital, Air Force Medical University, Xi’an, China; 6 Basic Medicine School, Air Force Medical University, Xi’an, China; 7 Department of Respiratory and Critical Care Medicine, Shenzhen General Hospital, Shenzhen University, Shenzhen, China; 8 State Key Laboratory of Cancer Biology, Department of Immunology, Air Force Medical University, Xi’an, Shaanxi 710032 China

**Keywords:** Lung cancer, Engineered exosomes, Drug delivery, Brain metastasis, Blood-brain barrier

## Abstract

Brain metastasis (BM) is one of the leading causes of cancer-related deaths in patients with advanced non-small cell lung cancer (NSCLC). However, limited treatments are available due to the presence of the blood-brain barrier (BBB). Upregulation of lysophosphatidylcholine acyltransferase 1 (LPCAT1) in NSCLC has been found to promote BM. Conversely, downregulating LPCAT1 significantly suppresses the proliferation and metastasis of lung cancer cells. In this study, we firstly confirmed significant upregulation of LPCAT1 in BM sites compared to primary lung cancer by analyzing scRNA dataset. We then designed a delivery system based on a single-chain variable fragment (scFv) targeting the epidermal growth factor receptor (EGFR) and exosomes derived from HEK293T cells to enhance cell-targeting capabilities and increase permeability. Next, we loaded LPCAT1 siRNA (siLPCAT1) into these engineered exosomes (exo^scFv^). This novel scFv-mounted exosome successfully crossed the BBB in an animal model and delivered siLPCAT1 to the BM site. Silencing LPCAT1 efficiently arrested tumor growth and inhibited malignant progression of BM in vivo without detectable toxicity. Overall, we provided a potential platform based on exosomes for RNA interference (RNAi) therapy in lung cancer BM.

**Supplementary Information:**

The online version contains supplementary material available at 10.1186/s12951-024-02414-7.

## Introduction

Lung cancer is the leading cause of cancer-related deaths worldwide, resulting in approximately 350 deaths per day [1]. Brain metastases (BM) are the main cause of high mortality in lung cancer patients [2]. Lung cancer is also the most common primary tumor that metastasizes to the brain, accounting for 40-50% of cases, which is significantly higher than breast cancer (15-25%) and melanoma (5-20%) [3, 4]. While tyrosine kinase inhibitors (TKIs) targeting mutant epidermal growth factor receptor (EGFR) have improved the survival of patients with non-small cell lung cancer (NSCLC) in the last decade, NSCLC patients with mutated EGFR are more likely to spread to the brain than those with wild-type EGFR [5–7]. Despite the promising initial responses to TKIs, patients eventually develop acquired drug resistance [8]. As a result, patients often experience central nervous system (CNS) relapse and succumb to the disease. Although immune checkpoint inhibitors targeting the PD-1-PD-L1 axis have achieved success as a standard treatment for a variety of cancers, the occurrence of immune-related toxicities and tolerance in a significant number of patients limit its effectiveness in treating BM (only 17–44% of BM patients benefit from this treatment) [9]. Therefore, there is an urgent need to develop more effective therapeutic strategies to suppress lung cancer brain metastases. Nevertheless, one major obstacle to delivering effective therapeutic to the tumor sites is the blood-brain barrier (BBB).

Exosomes are nano-scale (30-200 nm) extracellular vesicles that can be secreted into the bloodstream by various types of cells, exerting biological functions remotely. Exosomes play an important role in tumorigenesis, cancer metastasis, and drug resistance by remodeling the tumor microenvironment through the transfer of exosomal contents to recipient cells [10]. Exosomes have been shown to be a natural carrier with low immunogenicity and high compatibility, making them an ideal tool for transporting therapeutic reagents for cancer treatment. For example, in a mouse model of brain metastases of breast cancer, CXCR4/TRAIL-enriched exosomes enhanced the anti-tumor efficacy of chemotherapy, while exosomes-based delivery of cPLA2 siRNA and metformin targeting intracranial xenografts of glioblastoma suppressed the energy metabolism of tumor cells and exerted cell growth inhibitory effects [11, 12]. However, tumor treatment based on the delivery of exosome is not sufficiently accurate or efficient. Therefore, targeting antibodies or peptides are often anchored on the exosome surface to introduce a cell-targeting feature and increase permeability [13].

Lysophosphatidylcholine acyltransferase 1 (LPCAT1), a cytosolic enzyme that converts lysophosphatidylcholine into phosphatidylcholine, is highly expressed in multiple cancer types, including lung cancer [14], glioblastoma [15], endometrial cancer [16], and esophageal carcinoma [17]. LPCAT1 has been shown to significantly promote brain metastases of lung cancer by upregulating the PI3K/AKT/MYC pathway, making it a promising target for treating BM in lung cancer patients. Gene silencing of LPCAT1 remarkably reduced tumor cell proliferation in vitro and attenuated BM in vivo [14].

**Fig. 1:**
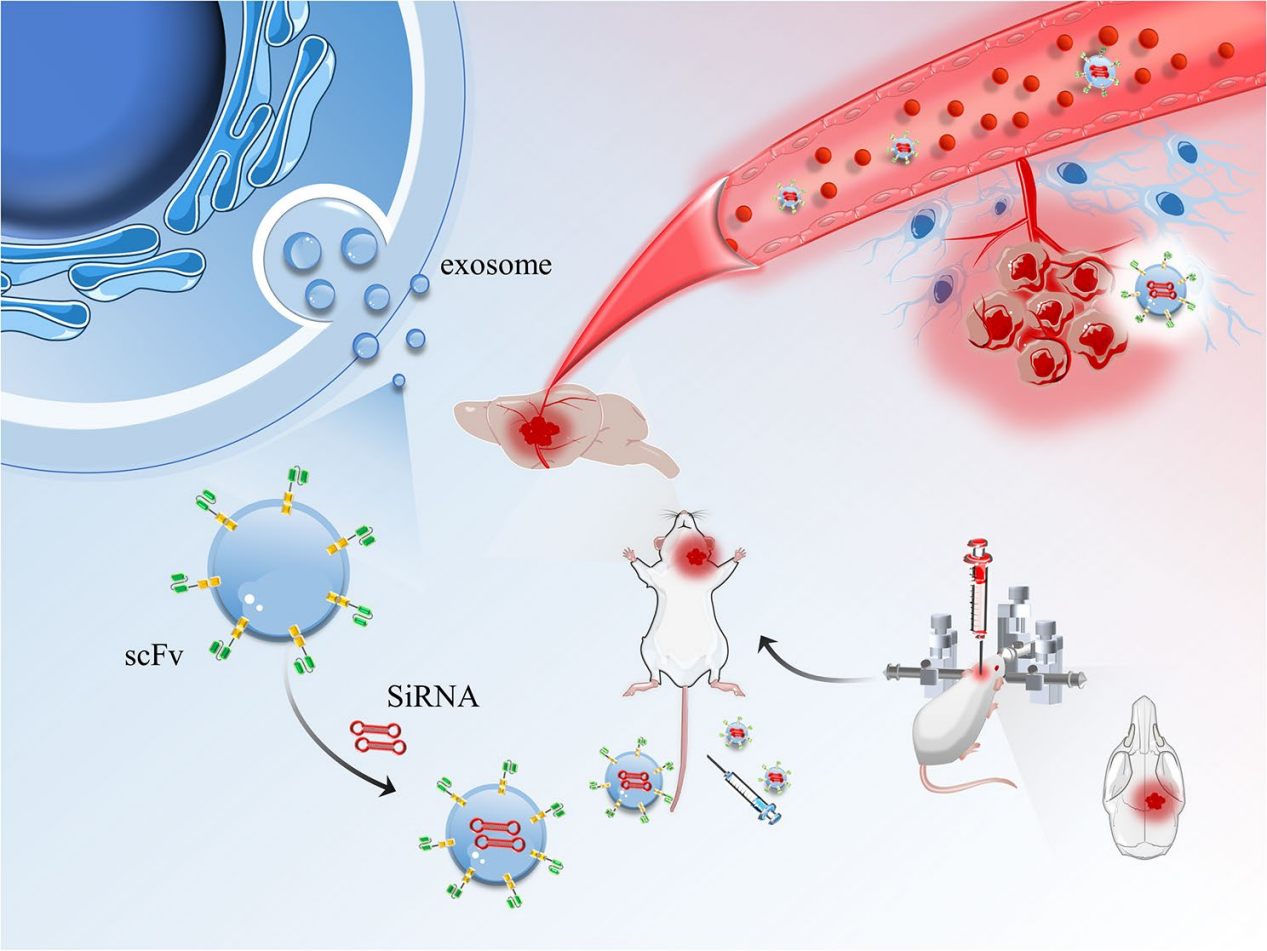
A Schematic illumination of exo^scFv^ construction, mouse model of lung cancer BM and RNAi treatment

Previously, we developed an EGFR-specific single-chain antibody fragment (scFv), which was used as a cell-targeting tool to deliver siRNA to EGFR-positive xenografts in a mouse model and to reverse drug resistance of EGFR-TKI [18]. We also synthesized targeting nanoparticles based on this scFv and demonstrated its feasibility as a contrast agent for MRI in vivo [19]. In this study, we designed a fusion protein consisting of scFv and exosomal surface protein lamp2b, which was expressed on the surface of exosome derived from HEK293 cells to enable cell targeting and therapeutic functions. We then encapsulated the engineered exosomes armed to EGFR with LPCAT1 siRNA (siLPCAT1) and intravenously injected them into a mouse model of lung cancer BM (Fig. 1). The engineered exosomes (exo^scFv^) displayed high intracranial permeability and delivery efficiency to the brain tumor. Importantly, our data showed that LPCAT1 siRNA-loaded scFv-coated exosomes (exo^scFv/siLPCAT1^) exerted potent anti-tumor effects in metastatic brain tumors from lung cancer.

## Materials and methods

### Data retrieval and preprocessing

We employed a single-cell RNA sequencing (scRNA-Seq)-based profiling method to quantitatively determine the cell types and states within NSCLC. We firstly searched in the Gene Expression Omnibus (GEO) and finally included one cohort (GSE131907, Ahn M et al. [20]) with scRNA-seq data of 58 tissue samples from 44 patients, consisting of 11 normal lung tissues, 11 primary tumor tissues, 10 brain metastases and other tissues. The clinical information of the GSE131907 dataset was available on the GEO database (https://www.ncbi.nlm.nih.gov/geo/). The scRNA-Seq data passed the quality control were then input into the Seurat R package (package v4.0.1) to obtain the unsupervised clustering based on the first 20 principal components of the top 2000 most variable genes among the whole-genome. UMAP (“RunUMAP”function) was used for the visualization of clustering [21].

### Plasmid construction

Genes encoding the lamp2b and anti-EGFR scFv-lamp2b fusion protein were synthesized by AuGCT Technologies, Inc (Beijing, China). The scFv-lamp2b fusion protein, in which the scFv and lamp2b are linked via a flexible (GGGGS)3 peptide, contained a 6 × His.tag at the C-termini of lamp2b. To generate expression constructs of exo^scFv^, scFv gene fragments were cloned into the pcDNA3.1(-) vector between NheI and XhoI restriction enzyme sites. The generated expression constructs were confirmed by DNA sequencing (LC-Bio, Inc, Hangzhou, Zhejiang, China).

### Cell culture and preparation

HEK293T cells and PC9 cells were purchased from Shanghai Institutes for Biological Sciences (Chinese Academy of Sciences, Shanghai, China). HEK293T cell line is commonly used to express exogenous genes. Thus, scFv-modified exosomes derived from HEK293T cells are feasible vesicles for delivering therapeutic molecules [22]. HEK293T cells were cultured in Dulbecco’s modified Eagle’s medium (DMEM; Gibco BRL, USA) with 10% fetal bovine serum (FBS, Biological Industries, Beit Ha’emek, Israel), 100 U/mL of penicillin, and 100 U/mL of streptomycin. Human PC9 cells were cultured in 1640 medium (Gibco, USA) with 10% FBS and 1% penicillin-streptomycin. All cells were incubated at 37 °C in a 5% CO2 atmosphere. A luciferase plasmid was constructed based on the pLenti 6.3 lentiviral vector, and the methods of establishing PC9 clones stably expressing luciferase were similar to those described before [23].

### Exosomes production, isolation, characterization and half-life

To generate exosomes that highly express surface anti-EGFR scFv-lamp2b (exo^scFv^) and lamp2b (exo^ctrl^), HEK293T cells were transfected with the expression constructs using transfection reagent lipofectamine 2000 (Invitrogen, Carlsbad, CA, USA). Six hours later, the cell culture medium was replaced with exosome-free medium and incubated for another 48 h. The cell culture supernatants were then collected and centrifuged at 500 g for 10 min, 2,000 g for 10 min, and 10,000 g for 30 min, respectively, to remove cells, residual cell debris, and larger extracellular vesicles in sequence. After being filtered by 0.22 μm filters (Millipore), the supernatants were centrifuged at 140,000 g for 2 h using an SW32 TI rotor in an Optima XE-100 ultracentrifuge (Beckman Coulter). The isolated exosome precipitates were re-suspended in EV-guardTM storage buffer (system biosciences, SBI, US, cat# EXSBA-1) and stored at − 80 °C. The exosomes were negatively stained by 4% uranyl acetate and observed using a transmission electron microscopy (TEM: JEM-1230, JEOL Ltd., Tokyo, Japan). The particle size distribution of the exosomes was measured by the nanoparticle tracking analysis (NTA) using a ZetaView Particle Metrix. Half-life (τ1/2) was evaluated using equation, τ1/2 = ln2/k = (t2-t1)*ln2/(lnC1-lnC2), (C1, fluorescence intensity at time 1 (t1); C2, fluorescence intensity at time 2 (t2)).

### Western blot analysis

To validate whether the fusion protein was successfully expressed in 293T cells and integrated into the exosome membranes, we performed western blot using anti-His antibody. Total protein of isolated exosomes or cells was extracted in RIPA Lysis Buffer (Solarbio, China) at 4 °C for 15 min. The protein concentration was determined using a BCA protein assay kit (Solarbio, China). About 30 µg of protein was separated on 10% SDS-PAGE gels, and then transferred to nitrocellulose membranes (Millipore). The membranes were blocked with 3% BSA, and then incubated with anti-His-tag (1:5000, Abmart, China, #M20001), and anti-β-actin (1:4000, Sigma, St Louis, MO, USA) at 4 °C overnight. Anti-CD63 (1:2000, Proteintech, Wuhan, Hubei, China, #25682-1-AP), anti-GM130 (1:2000, Proteintech, #11308-1-AP), anti-TSG101 (1:2000, Proteintech, #28283-1-AP) were also used to characterize the exosomes. anti-LPCAT1 (1:2000, Proteintech, #16112-1-AP), anti-EGFR (1:5000, Proteintech, #18986-1-AP). This procedure was followed by adding HRP-conjugated secondary antibody (1:10000, Cell Signaling Technology, Beverly, MA, USA) and ECL reagents (Solarbio, Beijing, China). The bands were visualized and recorded using the Tanon 5500 imaging system.

### Exosomes labeling and targeting in vitro

For in vitro experiments, the PKH76 fluorescent dye (Umibio, Shanghai, China) was used to label exosomes according to the manufacturer’s instructions. PC9 cells were seeded in confocal dishes, and co-incubated with PKH67-labeled exo^scFv^/exo^ctrl^ for 3 and 6 h. The cells were then fixed in 4% (w/v) paraformaldehyde in PBS for 15 min, and the nuclei were stained with 1.5 µg/mL of 4,6-diamidino-2-phenylindole dihydrochloride (DAPI, Sigma-Aldrich, USA) at room temperature for 5 min. After washing in PBS, the intracellular distribution of exosomes was visualized with a fluorescence microscope (Nikon, Tokyo, Japan).

### Preparation of siRNA-loaded exosomes and encapsulation efficiency

Protein concentration of exosomes was measured using a BCA assay. Exo^scFv^/exo^ctrl^ (100 µg with a protein concentration of 1 µg/µL) was mixed with 5 OD of siRNA at 4 °C for 30 min (The total volume was no more than 400 µL). The mixtures were then added into electroporation cuvettes (cap size: 2 mm). Electroporation was performed using the Gene Pulser Xcell Electroporation System (Bio-Rad, Hercules, CA, USA) and the electroporation condition was as followed: 350 V, 150 mA, 2 pulses. After electroporation, the exosomes were cooled on ice for 30 min.

To calculate the encapsulation efficiency of siRNA, various concentration of FAM labeled siRNA was diluted in DEPC water. A fluorescence standard curve was plotted based on different concentrations (0, 8, 16, 32, 64, 128, 256 and 320 ng/ml) of FAM-siRNA. After electroporation, the exosomes were centrifuged and resuspended in fresh PBS. The fluorescence intensity of free FAM-siRNA in the supernatant was detected and the concentration of free FAM-siRNA (C_free siRNA_, ng/ml) was calculated. Encapsulation Efficiency (EE%) = (W_siRNA_ - C_free siRNA_ × V_free siRNA_) / W_siRNA_ ×100%. W_siRNA_, total weight of siRNA, V_free siRNA_, volume of siRNA.

### Quantitative realtime PCR

Total RNA was extracted from cells using TRIzol reagent (Invitrogen, Carlsbad, CA, USA). cDNA was synthesized using a Prime Script qRT-PCR Kit (Takara Bio Inc., Japan). The expression level of LPCAT1 was tested by qPCR (Bio-Rad CFX Manager 3.1). Correlation data were calculated based on the ΔΔCT of the target gene and the internal reference actin. The primer sequences used were as follows (F, forward; R, reverse): LPCAT1-F: ACATCCCGATCTGGGGAACT; LPCAT1-R: GGCCACTTTCCGTTGGACT. actin-F: CCTGGGCATGGAGTCCTGTG; actin-R: TCTTCATTGTGCTGGGTGCC.

### Cell apoptosis and proliferation assay

The effect of different exosome formulations on cell apoptosis of PC9 was assessed by using the AnnexinV-FITC/PI kit (Bestbio, Shanghai, China). Cell proliferation-associated ki67 was evaluated by FCM assay using APC antihuman Ki-67 antibody (Biolegend, San Diego, USA). Flow cytometry data were analyzed by Flow Jo software. Cell Counting Kit-8 (CCK-8) assay was also performed to determine the effect of exosomes on cell proliferation. All procedures were according to the manufacturer’s instruction.

### Animal experiments

All experimental procedures involving animals were conducted under a protocol reviewed and approved by the Ethics Committee of Air Force Medical University. 6-week-old of male BALB/C nude mice (18–20 g) were purchased from Vital River Laboratory Animal Technology Co., Ltd, Beijing, China. To evaluate the distribution of scFv modified exosomes, we firstly establish lung cancer tumor-bearing mice models, 2 × 10^6^ luciferase expressing PC9 cells were injected subcutaneously in the thigh of BALB/c nude mice. Approximately one week later, the tumor masses were visible to the naked eye. Nude mice were randomly assigned to three groups (PBS group, exo^ctrl^ group, exo^scFv^ group, *n* = 3). Every 200 µg exosomes (1 µg/µL) were then stained with 200 µl 1,1’-Dioctadecyl-3,3,3’,3’-Tetramethylindodicarbocyanine, 4-Chlorobenzenesulfonate Salt (Did, Beyotime, Shanghai, China) (20 µM) at 37 °C for 10 min, followed by centrifugal isolation as described above. 200 µg DiD-labelled exo^scFv^/exo^ctrl^ (1 µg/µL) were injected via the tail vein at a dosage of 10 mg/kg. The distribution of the exosomes in different organs were then analyzed by the IVIS imaging system (PerkinElmer, life sciences, USA). Identical illumination settings were used as follows: ex: 620 nm/em: 670 nm, Filter Position 5, 10 cm field of view, Binning factor of 4, 2 s exposure time.

Lung cancer BM model was established by intraparenchymally injecting 5 × 10^5^ luciferase expressing PC9 cells into striatum of BALB/c nude mice [24]. Briefly, the mice were anesthetized and placed into a stereotactic apparatus (RWD, Shenzhen, China). A small hole was drilled 2 mm lateral and 1 mm anterior to the bregma. PC9 cells were then injected into the right hemisphere at a depth of 3 mm using a 10 µl Hamilton syringe with a 30-gauge needle. The injection was conducted at a constant speed for over 2 min followed by an additional 2 min pause before removing the needle. Then the scalp was stitched. One week later, the tumors in mice brain were observed after injection of 150 mg/kg Dluciferin. Nude mice were randomly assigned to three groups (PBS group, exo^ctrl^ group, exo^scFv^ group, *n* = 3). 200 µg DiD-labelled exo^scFv^/exo^ctrl^ (1 µg/µL) were injected via the tail vein as well, at a dosage of 10 mg/kg. Penetration of exosomes was assessed by the IVIS imaging system.

To evaluate anti-tumor effects, mice with BM tumor were randomly divided into three groups (*n* = 5) and injected with siLPCAT1-loaded exo^scFv^/exo^ctrl^ (exo^scFv/siLPCAT1^/exo^scFv/siNC^, 1 µg/µL) or PBS via the tail vein every 3 day for a total 4 times, at a dosage of 5 mg/kg. The tumor location and volume in brain was monitored by detecting bioluminesence after injecting 150 mg/kg luciferin. Identical illumination settings were used as follows:ex: block/em: open, 10 cm field of view, Binning factor of 4, 0.5 s exposure time.

### Immunofluorescence assay

Mice brains were fixed in 4% paraformaldehyde for 15 min and carefully frozen in OCT freezing media then dissected into 8 μm thick slices. Cell nuclei were stained by DAPI (Invitrogen). The fluorescence of DiD-labelled exosomes was observed by EVOS FL Auto 1 Cell Imaging System. To evaluate the expression of LPCAT1 and Ki67 in mice brains, sections were incubated with the respective primary antibodies (Anti-LPCAT1 (Proteintech), anti-Ki67 (Cell Signaling Technology, Danvers, MA, USA)) at room temperature for 4 h. To detect apoptotic tumor cells within brain metastases after treatment, sections were stained with in situ Tunel kit (Roche, Mannheim, Germany).

### In vivo safety evaluation

Ten male BALB/c nude mice were randomly divided into two groups. One group received intravenous injection of 20 mg/kg exo^scFv/siLPCAT1^every other day for one week and the other group was treated with PBS as control. Mice were then sacrificed to harvest the tissues and blood samples at 24 h after the last administration. The whole blood samples were stored at room temperature for 2 h and centrifuged (4,000 g) at 4 °C for 10 min. The serum biochemical parameters including liver enzymes, and renal function were measure by automatic biochemical analyzers (Chemray 800 and Chemray 240, Rayto, China). Major organs, including heart, liver, spleen, lung and kidney, were fixed with 4% paraformaldehyde, embedded with paraffin, and then stained with hematoxylin-eosin (H&E). Tissue sections were also investigated for LPCAT1 expression by immunohistochemistry (IHC) to evaluate whether the exo^scFv/siLPCAT1^ alter LPCAT1 expression in major organs.

### Statistical analysis

All Data are presented as the mean ± standard deviation. All statistical analyses were conducted with GraphPad Prism 8.0. The statistical significance values between two groups were determined by the *Student t* test. One-way ANOVA was used for comparing the differences between groups. Probability values < 0.05 were considered statistical significance.

## Results

### LPCAT1 was highly upregulated in malignant cells of lung cancer brain metastases

**Fig. 2:**
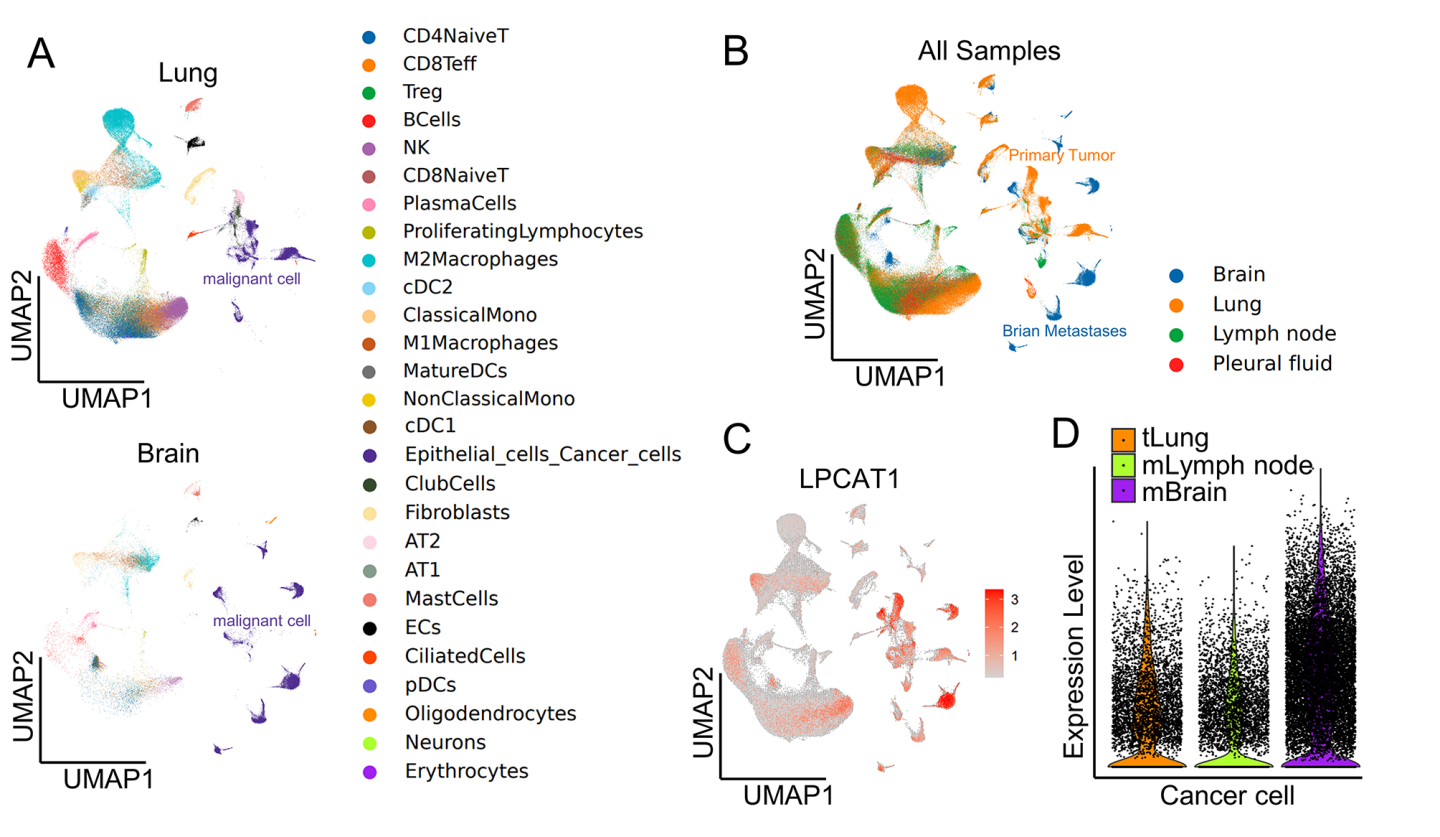
LPCAT1 expression patterns identified by scRNA seq analysis. **(A)** UMAP plot of cell subsets colored based on the major cell lineages and histological types including primary lung tumor, brain metastases tissues from GSE131907 dataset. **(B)** UMAP plot of all samples colored based on tissue types including lung, brain metastases, lymph node tissues and pleural fluid cells. **(C-D)**Average expression of LPCAT1 within lung tumor, brain metastases, lymph node tissues represented by UMAP plot and Violin Plot. tLung, lung tumor; mLymph node, lymph node metastases; mBrain, brain metastases

LPCAT1 was supposed to play a pro-tumoral role in NSCLC by promoting lung cancer cell proliferation and migration [14]. To further understand the heterogeneity and meaning of LPCAT1 expression in NSCLC primary tumors and metastatic lesions, we obtained and analyzed the lung adenocarcinoma brain metastases single cell transcriptome sequencing dataset (GSE131907). After data processing and annotation, uniform manifold approximation and projection (UMAP) profiles and 27 cellular subtypes were stratified. The 30,272 epithelial cancer cells were cataloged into 3 clusters with 0.8 resolution that primary lung cancer cells, metastatic brain tumor cells and metastatic lymph node cancer cells could optimally be separated by UMAP plot, suggesting significant heterogeneity in malignant cells between brain metastases and primary tumor (Fig. 2A-B). Moreover, the expression of LPCAT1 was found to be significantly elevated, specifically in BM samples, suggesting malignant cancer cells with upregulated LPCAT1 were prone to migrate to the brain (Fig. 2C-D).

### Isolation and characterization of anti-EGFR scfv-expressed exosomes

**Fig. 3:**
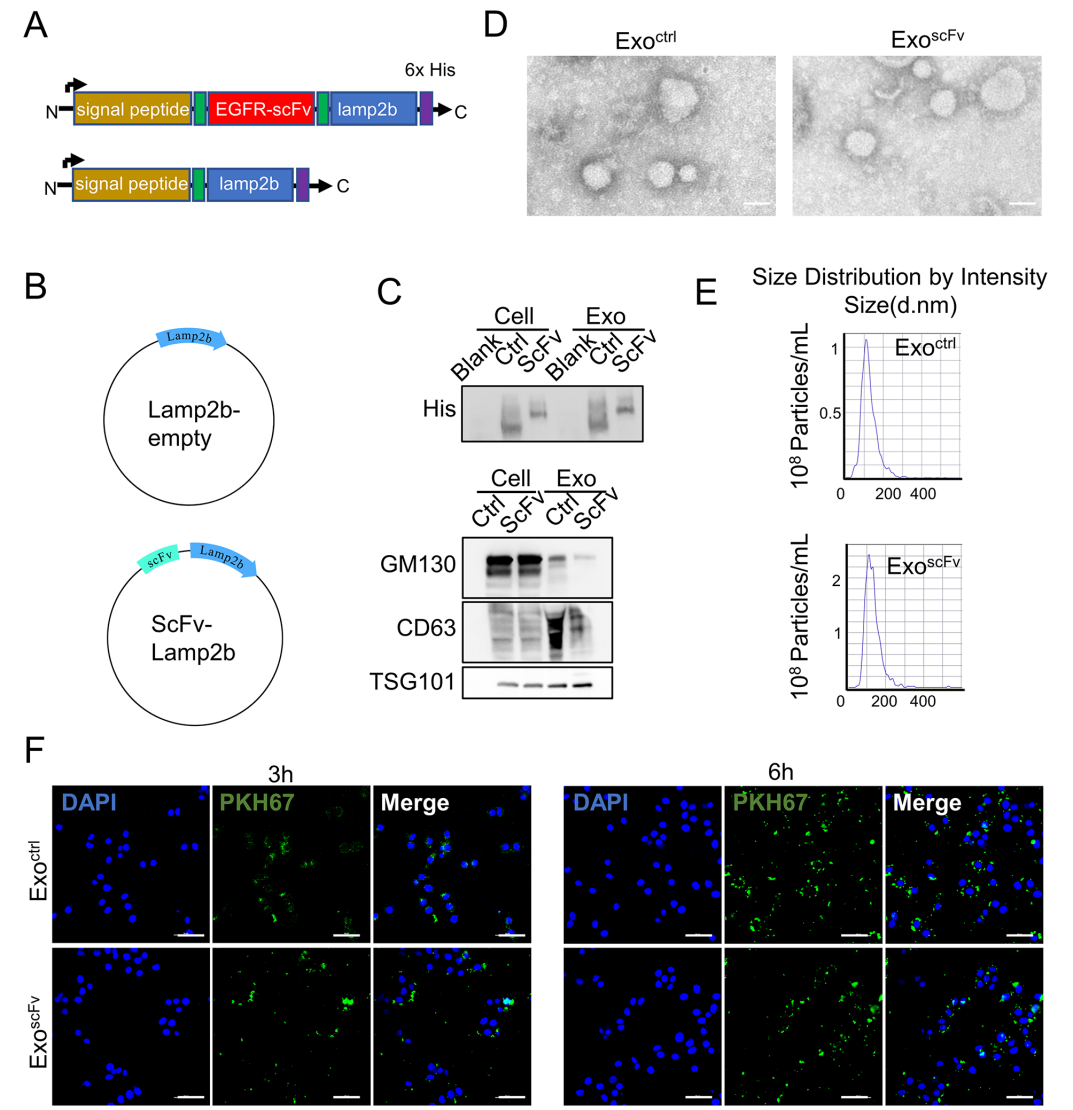
Exosome functionalized by EGFR specific scFv-Lamp2b fusion protein. **(A) **Schematic diagram of scFv-lamp2b and lamp2b fusion protein. **(B) **Plasmid structure of the genes of the fusion proteins. **(C) **Western blot analysis of the fusion protein expression in exosomes and their parental cells. **(D)** Representative TEM imaging of exo^scFv^ and exo^ctrl^. Scale bar = 100 nm. **(E)** Size distribution of exo^scFv^ and exo^ctrl^. **(F)** Internalization of exo^scFv^ and exo^ctrl^ visualized by confocal fluorescence imaging (Green: PKH76, Blue: DAPI). Scale bar = 20 μm

To confer targeting capability of exo^scFv^, we fused the anti-EGFR scFv to the exosomal membrane protein lamp2b, a protein abundantly expressed on exosomal membranes [25]. The coding sequence of anti-EGFR scFv was inserted into the fusion protein between the signal peptide and mature peptide coding sequence. A 6×his.tag was attached to the C terminus of lamp2b to confirm the expression of the recombinant protein and enable the location of exo^scFv^. A fusion protein containing merely lamp2b and his.tag was also constructed as the control exosomes referred to as exo^ctrl^ (Fig. 3A). The coding sequence was cloned into pcDNA3.1 (-) plasmids and then transfected into HEK293T cells (Fig. 3B). The exosomes were isolated and purified from the culture supernatants of 293T cells by ultracentrifugation. As shown in Fig. 3C, scFv-lamp2b was successfully expressed, and lamp2b was upregulated in the corresponding 293T cell and derived exosomes. The molecular weight of scFv-lamp2b-his.tag and lamp2b-his.tag fusion proteins was around 100 kd and 80 kd, respectively. The presence of exosomal markers CD63 and TSG101 was also confirmed by WB assay, while the Golgi apparatus marker GM130 was barely observed. The physical properties of modified exosomes were analyzed by TEM and NTA analysis. TEM was used to directly visualize the exosomes. Both exo^ctrl^ and exo^scFv^ had typical displayed saucer-like bilayer morphologies (Fig. 3D). NTA measurements showed that both kinds of exosomes had homogeneous particle size distribution with a mean diameter of 109.4 and 110.0 nm respectively, ranging from 30 to 150 nm in diameter. (Fig. 3E). In the stability test (Fig. S1), the size and polydispersity index (PDI) of incubation of exo^scFv^ remained at approximately 110 nm and 0.25, respectively, after five days of incubation in medium containing 10% FBS, indicating that exo^scFv^ could remain stable in the physiological environment.

### In vitro targeting of exo^scFv^

To evaluate the potential tumor targeting ability of exo^scFv^ in vitro, we labeled the exosomal part of exo^scFv^ and exo^ctrl^ with PKH76. As shown in Fig. 3F, a clear cytoplasmic fluorescent signal of PKH76 (green) in EGFR positive lung cancer PC9 cells was observed, which represents the uptake of exo^scFv^ and exo^ctrl^. A higher signal intensity was visible after 3 h of incubation in the exo^scFv^ group compared to the exo^ctrl^ group. The intensity difference between the two groups became more pronounced after 6 h of incubation, suggesting that the anti-EGFR scFv anchored on the extra-exosomal membrane enhanced the ability to recognize antigens on lung cancer cells and could rapidly guide exosomes to receiving cells and accelerate its internalization.

### In vivo distribution of exo^scFv^

**Fig. 4:**
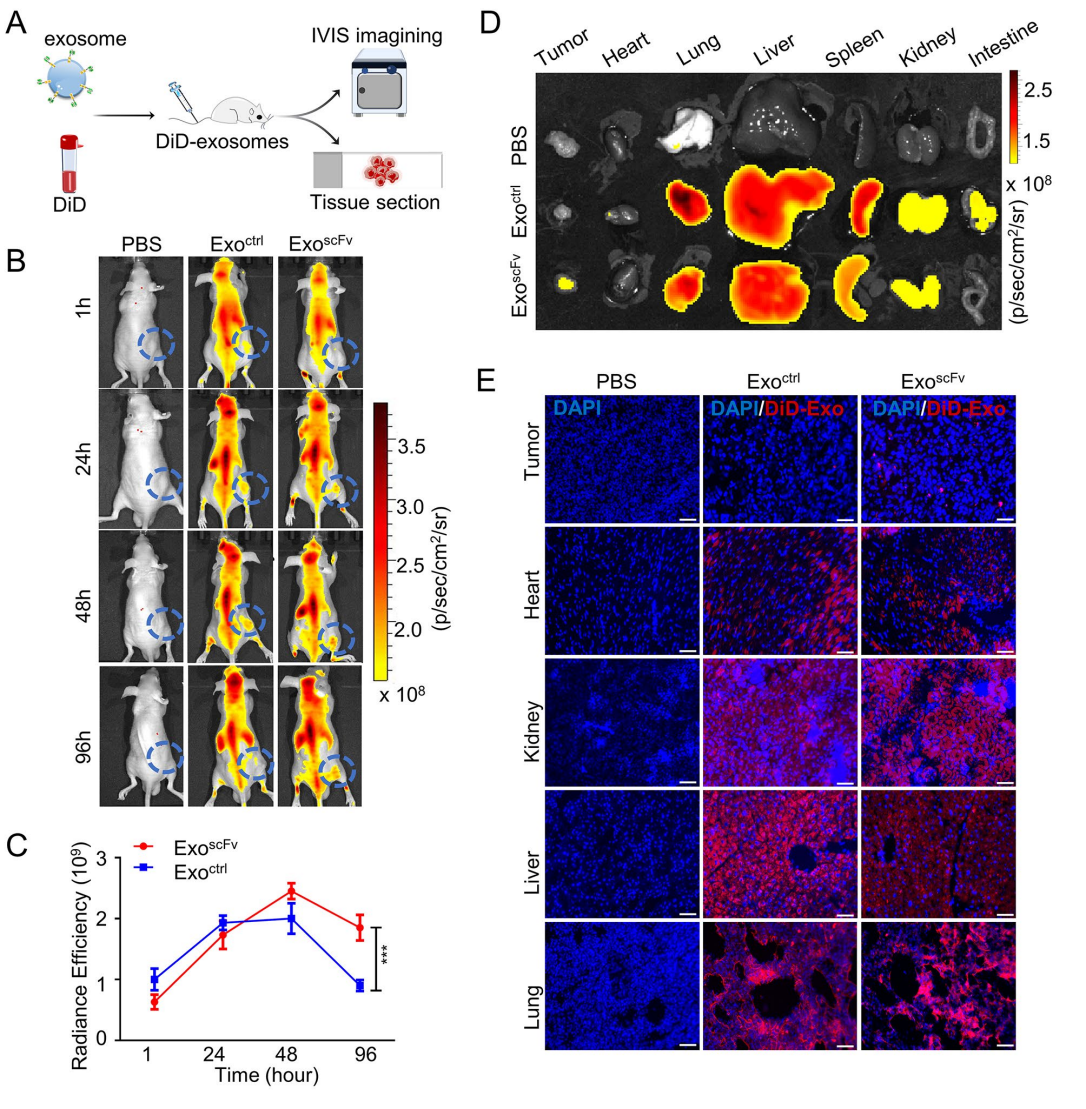
In vivo distribution of exo^scFv^ and exo^ctrl^ in PC9 tumor-bearing mice. **(A)** Schematic diagram of the experimental procedure. 200 µg exo^scFv^, 200 µg exo^ctrl^ or PBS were injected via the tail vein into PC9 tumor-bearing mice, *n* = 3 mice per group, exosomes dosage = 10 mg/kg. **(B)** Whole-body images of the exosome distribution. Images obtained at indicated time intervals after injection, *n* = 3 mice per group. **(C)** Changes in fluorescent intensity of tumor site over time, *n* = 3. **(D)** Ex vivo fluorescence imaging of the distribution of the DiD-labeled exosomes in organs at 96 h after injection. **(E)** Fluorescence images of tumor cryosections were obtained by confocal fluorescence imaging (Red: DiD, Blue: DAPI), Scale bar = 150 μm

To assess the tumor-targeting ability of exo^scFv^, we firstly established the tumor-bearing mice models, 200 µg DiD-labeled exo^ctrl^ or exo^scFv^ was injected via tail vein (Fig. 4A). The biodistribution of exosomes was monitored at 1 h, 24 h, 48 h, 96 h using IVIS. As shown in Fig. 4B, sufficient fluorescence could yield since 1 h post-injection and gradually diffused throughout the whole bodies, especially reticuloendothelial systems, such as liver, lung, and spleen. However, the fluorescence intensity in tumor masses of the control group began to weaken before 48 h after injection (Fig. 4C). In contrast, the tumor fluorescence intensity of the exo^scFv^ group reached the peak at 48 h. Moreover, the residual fluorescence intensity of the exo^scFv^ group at 96 h was two times higher than that of the exo^ctrl^ group. Notably, there were abundant exosomes detained in tumor verified by Ex vivo fluorescence imaging (Fig. 4C and Fig.S2). In addition, fluorescence imaging of frozen tissue sections showed DiD-labeled exosomes were detected mainly in the lung and liver, followed by the kidney, heart, and tumor, consistent with the results of in vivo imaging (Fig. 4E). Additionally, the half-life of exo^scFv^ in lung, kidney, liver and tumor is 14.51 h, 22.23 h, 32.89 and 71.37 h, respectively. These results suggested that EGFR-scFv modification effectively enhanced the tumor targeting of exosomes and prolonged the retention time of exosomes in the tumor.

**Fig. 5:**
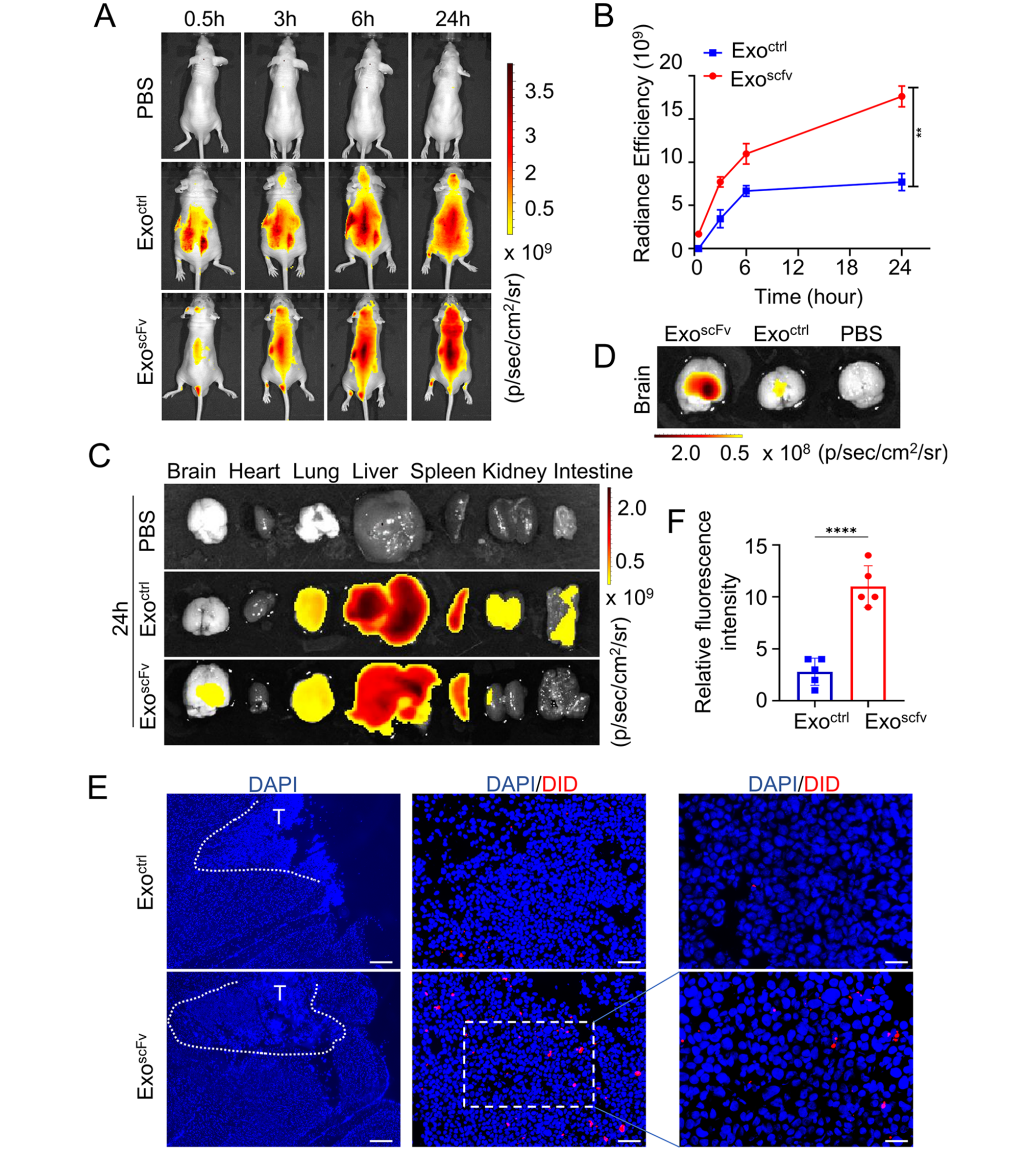
In vivo distribution of exo^scFv^ and exo^ctrl^ in PC9 BM model. **(A)** Whole-body images of the exosome distribution. Images obtained at indicated time intervals after injection, *n* = 3 mice per group, exosomes dosage = 10 mg/kg. **(B)** Changes in fluorescent intensity of brain part over time, *n* = 3. **(C)** Ex vivo fluorescence images of major organs at 24 h after injection. **(D)** Ex vivo fluorescence images of BM model.** (E)** Fluorescence images of tumor cryosections were taken by confocal fluorescence imaging (Red: DiD, Blue: DAPI). Scale bar = 750 μm (left), 150 μm (middle), 75 μm (right)^trl^. **(F)** Quantification of the fluorescence signal intensity in Fig. 5E. ****p < 0.0001, Data were expressed as mean ± SD, *n* = 5

To further evaluate the brain permeability and BM lesions targeting ability of exo^scFv^, we developed the animal BM model according to the previous method [26]. PC9 cells stably expressing luciferase were injected into the striatum of *BALB/c* nude mice by intraparenchymal implantation. Tumor growth was monitored by bioluminescence imaging. One week later, in vivo imaging showed that the BM model was successfully established as IVIS-positive foci were observed at the implantation sites (Fig. S3). The tumor-bearing mice were injected with DiD-labeled exo^ctrl^ or exo^scFv^ via tail vein, respectively. The biodistribution of exosomes was monitored at 0.5 h, 3 h, 6 h, 24 h using IVIS. 24-hour IVIS imaging was used in this study. As shown in Fig. 5A-B, imaging at 3 h after injection revealed a rapid accumulation of exo^scFv^ in the tumor site of mice brain, as the fluorescence intensity was much greater than that of the exo^ctrl^ group. Notably, there were abundant exosomes localized in the brain (Fig. 5C-D). Additionally, as shown in Fig. 5E-F, PC9 BM slices from the brain of the mice injected with exo^scFv^ exhibited significantly increased DiD signal intensity than those from the mice injected with exo^ctrl^, and no signal was observed in adjacent normal brain tissues. Importantly, the fluorescence difference of the two groups appeared earlier in the BM model, which might be due to the presence of the BBB, as exosomes have to enter the tumor site at a slower pace.

### Anti-tumor effect of exo^scFv/siLPCAT1^ in vitro

**Fig. 6:**
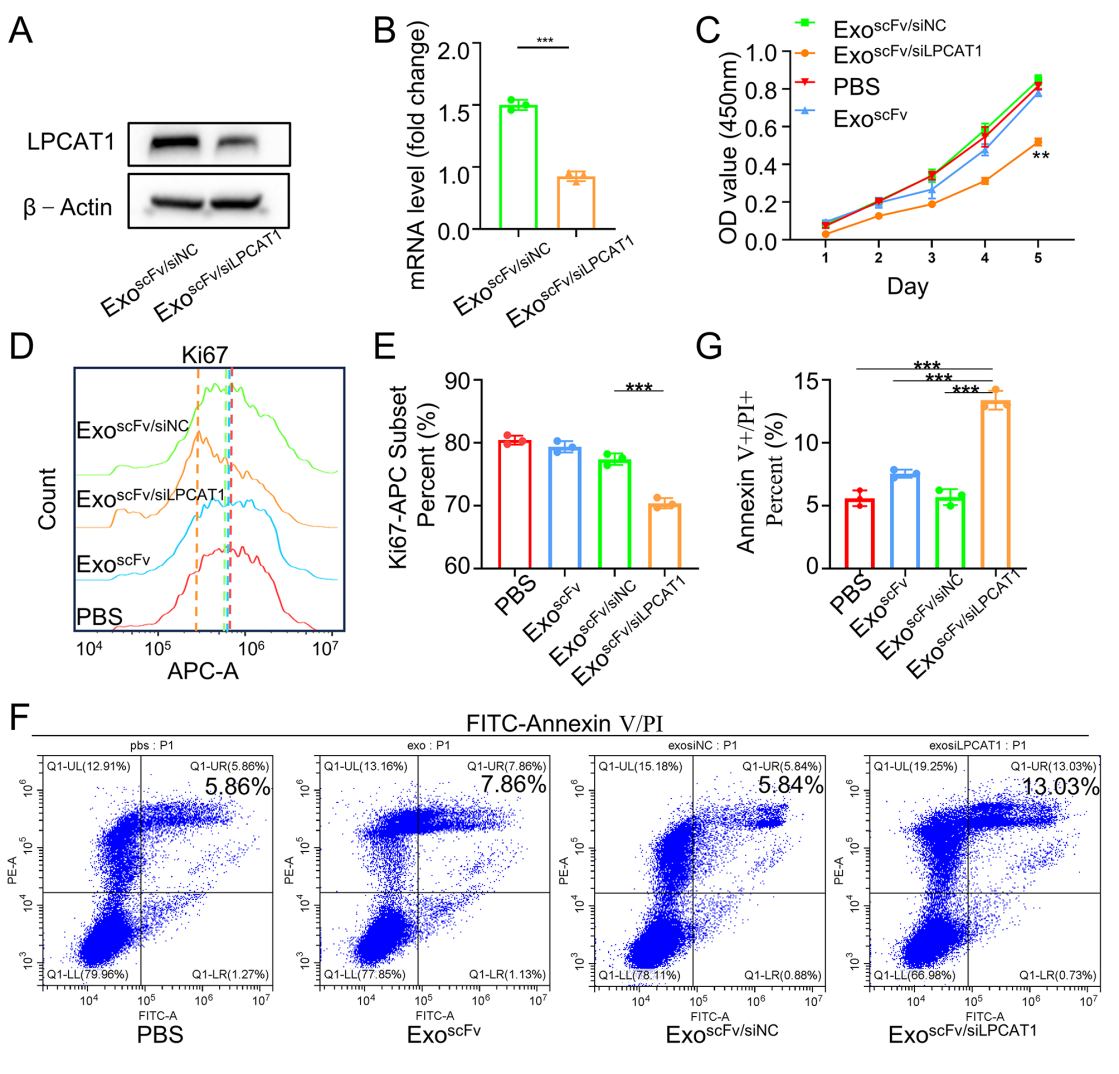
Anti-tumor effect of exo^scFv/siLPCAT1^ in vitro. **(A)** Western blot analysis of LPCAT1 protein expression in PC9 cells treated with exo^scFv/siLPCAT1^ or exo^scFv/siNC^. **(B)** The relative change in mRNA expression of LPCAT1 in PC9 cells treated with exo^scFv/siLPCAT1^ or exo^scFv/siNC^ (Data were expressed as mean ± SD. *n* = 3). **(C)** CCK8 assay of PC9 cells treated with PBS, exo^scFv^, exo^scFv/siLPCAT1^ or exo^scFv/siNC^, Data were expressed as mean ± SD. *n* = 3. **(D-E)** Flow cytometry assay of the expression of ki67 in PC9 cells treated with PBS, exo^scFv^, exo^scFv/siLPCAT1^ or exo^scFv/siNC^, color legend shared with panel C. **(F-G)** Flow cytometry assay of PC9 cells staining with AnnexinV-FITC/PI after being treated with PBS, exo^scFv^, exo^scFv/siLPCAT1^ or exo^scFv/siNC^, color legend shared with panel C. *p < 0.05, **p < 0.01, ***p < 0.001

To verify whether LPCAT1 could become a therapeutic target for NSCLC BM treatment, in this study, we firstly prepared exo^scFv^ loading with siLPCAT1. Under the optimum electroporation conditions, the encapsulation efficiency of exo^scFv^ for siLPCAT1 and siNC were 85.19 ± 1.21% and 86.07 ± 1.39%, respectively (Fig. S4). Then we evaluated the gene silencing effectiveness of LPCAT1 siRNA loaded exosomes (exo^scFv/siLPCAT1^) in PC9 cells. PC9 cells have much higher expression of EGFR than Beas2b lung epithelial cell (Fig. S5A). And exo^scFv/siLPCAT1^ resulted in a marked decrease expression of LPCAT1 in PC9 compared to the control (Fig. 6A-B, Fig.S5B). Next, we explored the role of exo^scFv/siLPCAT1^ in lung cell cancer proliferation and apoptosis by performing CCK8 and FCM assays. The CCK8 assay revealed that cell proliferation was significantly repressed in the exo^scFv/siLPCAT1^ group compared with the control (Fig. 6C). Similarly, the FCM assay showed that the proliferative activity of PC9 cells was significantly decreased in the exo^scFv/siLPCAT1^ group (Fig. 6D-E). The FCM assay also revealed that exo^scFv/siLPCAT1^-treated PC9 cells had a significantly higher late apoptotic rate (13.03 ± 0.73%) than exo^scFv/siNC^-treated cells (5.84 ± 0.88%) (*p* < 0.001), and only 7.86 ± 1.13% exo^scFv^ (*p* < 0.001) and 5.86 ± 1.27% PBS-treated cells (*p* < 0.001) were undergoing apoptosis (Fig. 6G-F).

### In vivo anti-tumor effect of exo^scFv/siLPCAT1^

**Fig. 7:**
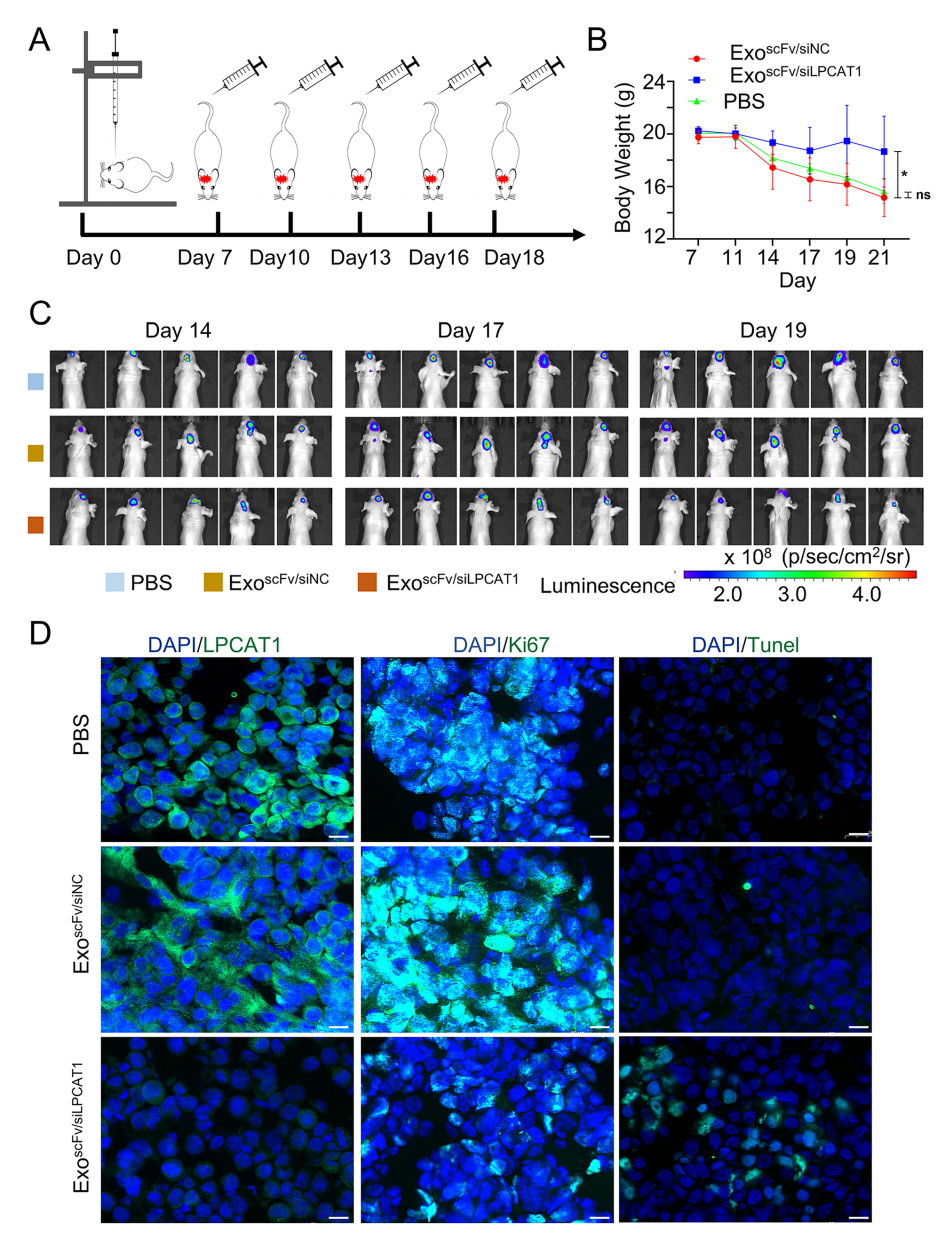
Anti-tumor effect of exo^scFv/siLPCAT1^ in vivo. **(A)** Schematic illumination of the experimental procedure. **(B)** Change of body weight of BALB/C nude mice treated with exo^scFv/siLPCAT1^, exo^scFv/siNC^ or PBS, *p < 0.05, ns, not significance, *n* = 5 mice per group. Exosomes dosage = 5 mg/kg. **(C)** Representative bioluminescent images of BM tissues of nude mice treated with exo^scFv/siLPCAT1^, exo^scFv/siNC^ or PBS at indicated time intervals after injection, *n* = 5 mice per group. **(D) **The signals of LPCAT1 (Green), ki67 (Green) and tunel (Green) in BMs sections were detected by confocal fluorescence imaging. Scale bar = 50 μm

We next explored whether EGFR specific scFv functionalized exo^scFv^ could efficiently deliver siLPCAT1 across BBB to the BM site of lung cancer in vivo. The schematic representation of the in vivo assay procedure was shown in Fig. 7A. The body weight of mice was recorded the day after injection, and the tumor growth was monitored by IVIS during the same period. As shown in Fig. 7B, mice in the exo^scFv/siLPCAT1^ treated group lost less body weight by the first week than the PBS or exo^scFv/siNC^-treated mice, and body weight loss became significant from the 19th day until the 21th day. We also visually testified that exo^scFv/siLPCAT1^ had a superior anti-tumor effect compared with the control group, as the average load of brain metastases measured photon flux in IVIS was significantly lower. The tumor in the mice treated with PBS or exo^scFv/siNC^ grew rapidly and emitted strong luminescence. The exo^scFv/siLPCAT1^ treated group displayed a significantly smaller area and weaker intensity of bioluminescence, and the quantitation revealed that tumor luminescent intensity at 19th day was approximately 4.51-folds lower in exo^scFv/siLPCAT^ than in the exo^scFv/siNC^ group (Fig. 7C, Fig. S6). To testify the silencing efficacy of siLPCAT1 in vivo, we further detected the expression of LPCAT1 in the BM site by immunofluorescence staining analysis. The expression of LPCAT1 in tumors from the exo^scFv/siLPCAT1^ group was significantly downregulated compared with those from the PBS and exo^scFv/siNC^ group (Fig. 7D). Meanwhile, to elucidate the cellular proliferation and apoptosis alteration of BM under the impact of LPCAT1 downregulation, the TUNEL and Ki67 staining were performed. As shown in Fig. 7D and Fig. S7A, the number of TUNEL positive cells were significantly increased in tumors from mice treated with exo^scFv/siLPCAT1^ compared with those from mice treated with PBS or exo^scFv/siNC^. Correspondingly, a significant decrease in the number of Ki67-positive cells were also detected in tumors from the exo^scFv/siLPCAT1^ group (Fig. 7D and Fig. S7B).

#### Safety evaluation of exo^scFv/siLPCAT1^

**Fig. 8:**
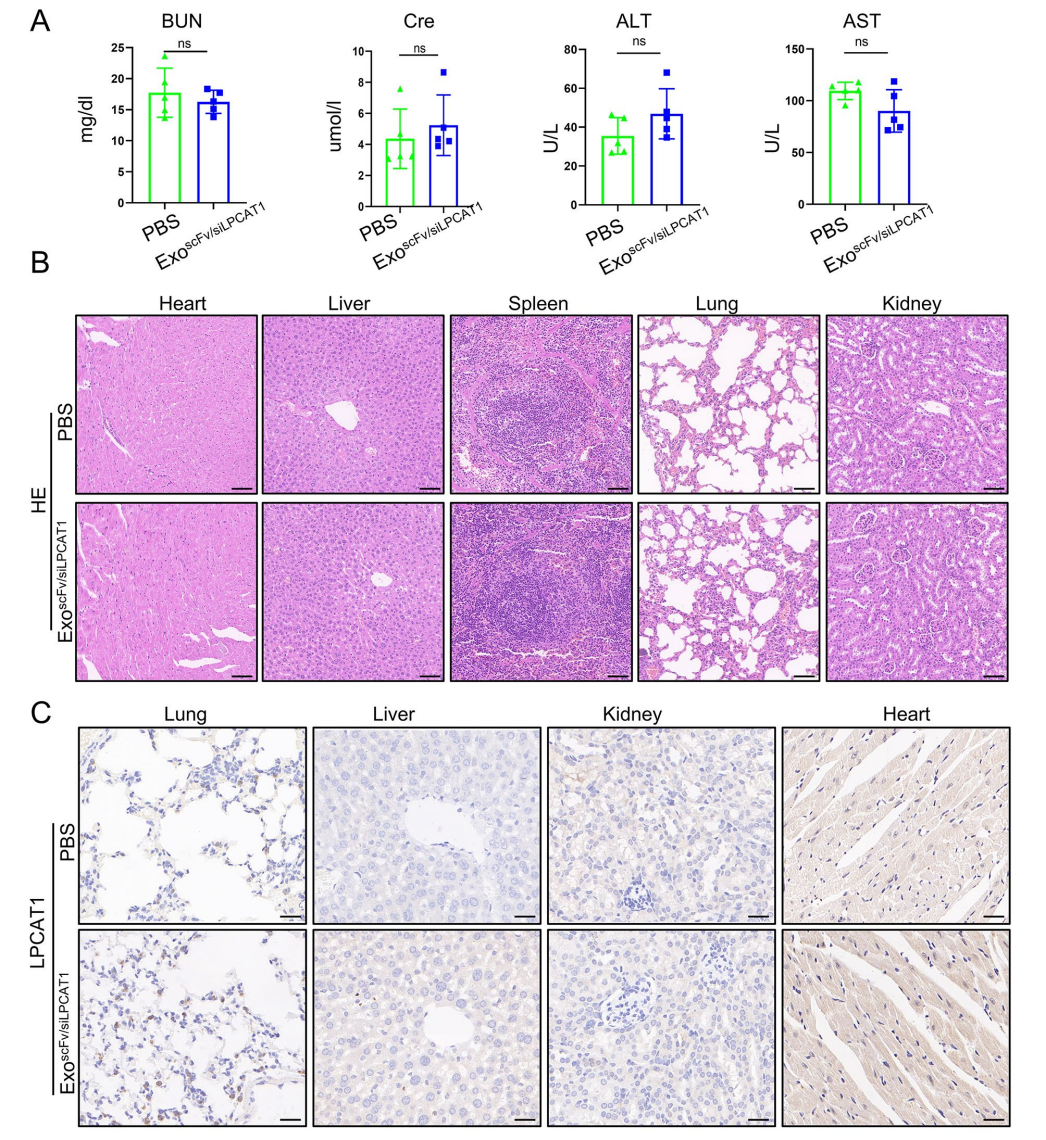
The systematic toxicity assessment of exo^scFv/siLPCAT1^. **(A)** Serum biochemical values of mice being treated with exo^scFv/siLPCAT1^ or PBS, *n* = 5 mice per group. Exosomes dosage = 20 mg/kg. ALT: alanine aminotransferase, AST: aspartate aminotransferase, BUN: blood urea nitrogen, CRE: creatinine. **(B)** Representative H&E staining of heart, lung, liver, kidney and spleen sections of mice being treated with exo^scFv/siLPCAT1^ or PBS. Scale bar = 100 μm. **(C)** IHC staining of LPCAT1 in lung, liver, kidney and heart. Scale bar = 50 μm

We investigated the toxicity of exo^scFv/siLPCAT1^ on mice and collected serum samples at a dosage of 20 mg/kg every other day for one week to measure aspartate aminotransferase (AST), alanine aminotransferase (ALT), blood urea nitrogen (BUN), and creatinine (CRE). As shown in Fig. 8A, no obvious hepatic or renal toxicity was observed in the exo^scFv/siLPCAT1^ treated group compared to the PBS treated group. Additionally, we examined hematoxylin and eosin (H&E)-stained sections of major organs, including the heart, lung, liver, spleen, and kidney from the exo^scFv/siLPCAT1^ and PBS treated groups. No evidence of structural damage was observed in the exo^scFv/siLPCAT1^ group as well (Fig. 8B). Meanwhile, there was no significant difference between the two groups in the expression of LPCAT1 in the tissues of the lung, liver, kidney, and heart as detected by IHC assay (Fig. 8C). The value of CRE of both PBS and exo^scFv/siLPCAT1^ group was less than 10 µmol/L the limits of the reference range, which may be due to reduced food intake caused by brain tumors. Above results indicated that high dosage of exo^scFv/siLPCAT1^ did not cause acute toxicity to major organs in nude mice.

## Discussion

Nanoparticles have been extensively researched as particulate drug carriers, and they have shown excellent results during the past decades. In cancer therapeutics, they offer exciting solutions for improving the efficacy of therapeutic reagents by prolonging their residence time within lesions and simultaneously overcoming side effects by minimizing normal tissue accumulation [27]. For example, albumin-bound nanoparticle (nab) paclitaxel exhibits enhanced paclitaxel tissue distribution and tumor penetration, and has become the most successful anti-tumor nanomedicine used in the clinic [28]. Nonetheless, for most nanomedicine, the potent toxicity (genotoxicity, immunotoxicity, cellular stress, inflammation) derived from their constituents still significantly limits their translational applications, and novel materials are required. Exosomes have been investigated for their therapeutic potential in pre-clinical studies. Exosomes tend to have different organ tropism based on their cells of origin, which could be utilized for organ-targeted delivery of therapeutic reagents [29]. For example, doxorubicin-loaded exosomes further reduced tumor growth with no apparent adverse effects observed with equipotent free drug [30]. Moreover, the exosome-based strategy alters drug intracellular trafficking by overcoming drug efflux systems. In a mouse model, exosomes used as carriers to deliver paclitaxel to multiple drug-resistant cancer cells showed increased drug cytotoxicity and potent anticancer effects [31]. However, a previous study also suggested that tumor-derived exosomes have tumor-targeting properties only when locally injected into the tumor site [32]. To more efficiently deliver their therapeutic payload to target cells, exosomes have been modified using two major approaches: exogenously modifying the surface of exosomes or genetically expressing targeting moieties in the original cells to produce active targeted exosomes. For instance, A33-positive exosomes could form a complex with A33 antibody-coated superparamagnetic iron oxide nanoparticles (SPIONs) and thereby gain targetability towards A33-positive colon cancer cells [33]. Upon conjugating with transferrin-modified SPIONs through transferrin-transferrin receptor interaction, TNF-α coated exosomes exhibited a prolonged circulation time and enhanced in vivo antitumor activity [34]. Exosomes surface can also be manipulated to display targeting peptides or antibodies, which are expressed as fusions with exosomal membrane-associated domains such as lamp2b or C1C2, to achieve tissue targeting. In Alzheimer’s disease research, RVG peptide expressed with lamp2b protein on the exosomal surface can introduce the target delivery of BACE1 siRNA into mice brain and demonstrate efficient gene silencing [25]. Anti-EGFR nanobodies fused with glycosylphosphatidylinositol (GPI)-anchor signal peptide enriched on the surface of exosomes increase the binding of exosomes to EGFR positive tumor cells [35]. CAR exosomes, released from EGFR or HER2 scFv-transduced CAR-T cells, induce potent antitumor inhibition in target cancer cell lines and solid tumor xenografts [36]. In the present study, we engineered HEK293T cells to express the fusion protein of scFv-lamp2b to produce tumor-targeting enhanced exosomes. This scFv has been demonstrated to efficiently deliver siRNA or SPOINs into EGFR positive lung cancer cells both in vitro and in vivo [18, 19]. Our in vitro results showed that the functionalized exo^scFv^ could bind to EGFR positive PC9 cells more efficiently than exo^ctrl^, and exo^scFv/siLPCAT1^ caused a significant loss in cell viability compared to the cells treated with the control.

Brain metastases (BM) occur in up to 30-50% of patients with advanced NSCLC, especially in patients with EGFR positive mutations, such as 21exon L858R [37]. Nevertheless, few targeted TKIs can efficiently pass the BBB, and neither can chemotherapies. Exosomes have the capacity to cross the BBB, yet modifying them with ligands specific to the lesion area rather than the whole brain will accelerate the therapeutic applications of exosomes in brain diseases. Therefore, we next sought to determine whether surface modification of exosomes with specific scFv will affect gene silencing efficiency in the brain. Several kinds of animal models have been used in researching cancer BM, including intraparenchymal implantation [24], intracardiac injection [38], intracarotid artery injection [39], and spontaneous formation [40]. Directly intracardiac injection of tumor cells is often associated with undesirable complications such as artery thromboembolism, and spontaneous formation of BM is often associated with lower odds of success. On the other hand, our purpose was not to explore the mechanism of lung cancer BM, but to verify whether the exo^scFv^ hold potential for targeting and suppressing intracranial after crossing blood-brain barrier. Therefore, we chose intraparenchymal implantation to construct the BM model by injecting lung cancer cells directly into the brain parenchyma of mice mounted on a brain stereotaxie apparatus. The results showed that we provided a stable method to display EGFR-specific scFv on the surface of exosomes. Exo^scFv^ exhibited accelerated BBB penetration and prolonged tumor accumulation in BM lesions compared to exo^ctrl^, indicating increased gene silencing efficacy and diminished adverse effects. However, xenograte tumors in brain were great burden to mice, especially to control groups, and resulted in poor performance status, like weight loss and hypoactivity. Thus, we shortened the observation period as much as possible.

The current standard of care for locally advanced or metastatic NSCLC includes targeted therapy, chemotherapy, radiotherapy, and immunotherapy, but drug resistance within the tumor has crippled these treatments. RNAi technology (RNAi), especially siRNA, could be an alternative to control tumor growth by specifically silencing oncogenes [41]. However, naked siRNA usually lacks specificity, biocompatibility, and stability in blood circulation [42]. To preserve the integrity and functionality, siRNA has been encapsulated in carriers, including various nanoparticles, to avoid degradation from nucleases. In addition, siRNA-loaded nanoparticles have been conjugated with antibody or peptide ligands targeting specific biomarkers in tumors to improve specificity [43]. LPCAT1 was upregulated in NSCLC and was associated with poor prognosis of patients, partly by promoting CNS metabolism [14]. In our study, a great antitumor effect was observed after treatment of EGFR positive lung cancer cells with exo^scFv/siLPCAT1^ compared to exo^scFv/siNC^ both in vivo and in vitro. Importantly, our method is safe to deliver bioactive cargos, and no signs of systemic toxicity or side effects were observed, probably because no chemical modification was involved.

## Conclusion

In the present study, we demonstrated a method for preparing EGFR-specific scFv-anchored exosomes that showed enhanced anticancer activity. The scFv, which was C-terminally fused to lamp2b via genetic engineering, is an ideal tool for delivering therapeutic and diagnostic reagents to tumor sites. The EPR effect of exosomes and active targeting properties of scFv enable exo^scFv^ to gain favorable targeting properties. The retention time of exosomes in subcutaneous tumors was significantly prolonged by scFv modification. Moreover, our exo^scFv^ successfully transported LPCAT1 siRNA across the BBB to targeted BM sites and demonstrated potent antitumor efficacy with negligible toxicity. Taken together, our results highlight that the exosome-based EGFR targeting siRNA delivering system is a promising therapeutic strategy for BM of lung cancer.

## Electronic supplementary material

Below is the link to the electronic supplementary material.

Supplementary Material 1

Supplementary Material 2

Supplementary Material 3

Supplementary Material 4

## Data Availability

No datasets were generated or analysed during the current study.
