# Correction: Anti-EGFR ScFv functionalized exosomes delivering LPCAT1 specific siRNAsfor inhibition of lung cancer brain metastases

**DOI:** 10.1186/s12951-026-04714-6

**Published:** 2026-06-30

**Authors:** Jun Jiang, Yuan Lu, Jie Chu, Xiao Zhang, Chao Xu, Shaojie Liu, Zhuo Wan, Jiawei Wang, Lu Zhang, Kui Liu, Zhenhua Liu, Angang Yang, Xinling Ren, Rui Zhang

**Affiliations:** 1 Department of Health Service, Base of Health Service, Air Force Medical University, Xi’an, China; 2 Department of Respiratory and Critical Care Medicine, Zhongda Hospital, Southeast University, Nanjing, 210009 China; 3 State Key Laboratory of Cancer Biology, Department of Biochemistry and Molecular Biology, Air Force Medical University, Xi’an, China; 4 Department of Urology, Xijing Hospital, Air Force Medical University, Xi’an, China; 5 Department of Hematology, Tangdu Hospital, Air Force Medical University, Xi’an, China; 6 Basic Medicine School, Air Force Medical University, Xi’an, China; 7 Department of Respiratory and Critical Care Medicine, Shenzhen General Hospital, Shenzhen University, Shenzhen, China; 8 State Key Laboratory of Cancer Biology, Department of Immunology, Air Force Medical University, Xi’an, 710032 Shaanxi China

**Correction: Journal of Nanobiotechnology (2024) 22:159**

10.1186/s12951-024-02414-7

The authors identified an inadvertent image duplication in Figure 7D (specifically, the immunofluorescence images for Ki67). This unintentional error occurred during the final figure assembly process. Due to the large volume of image files generated for this study, the incorrect representative image was mistakenly inserted into the panel.

For completeness and transparency, the old incorrect and correct versions are displayed below.

Incorrect Fig. 7

**Figure:**
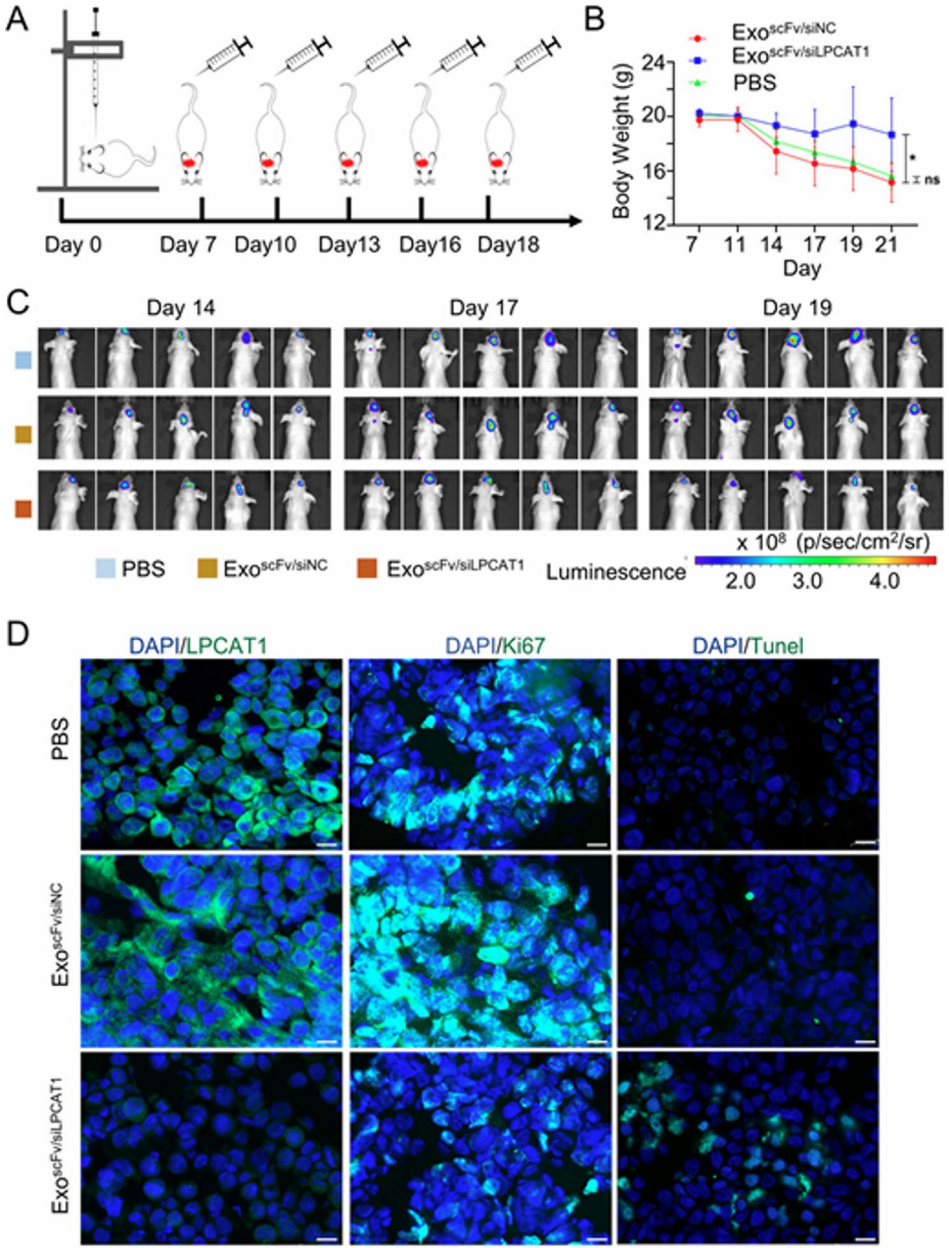

Correct Fig. 7.

**Fig. 7:**
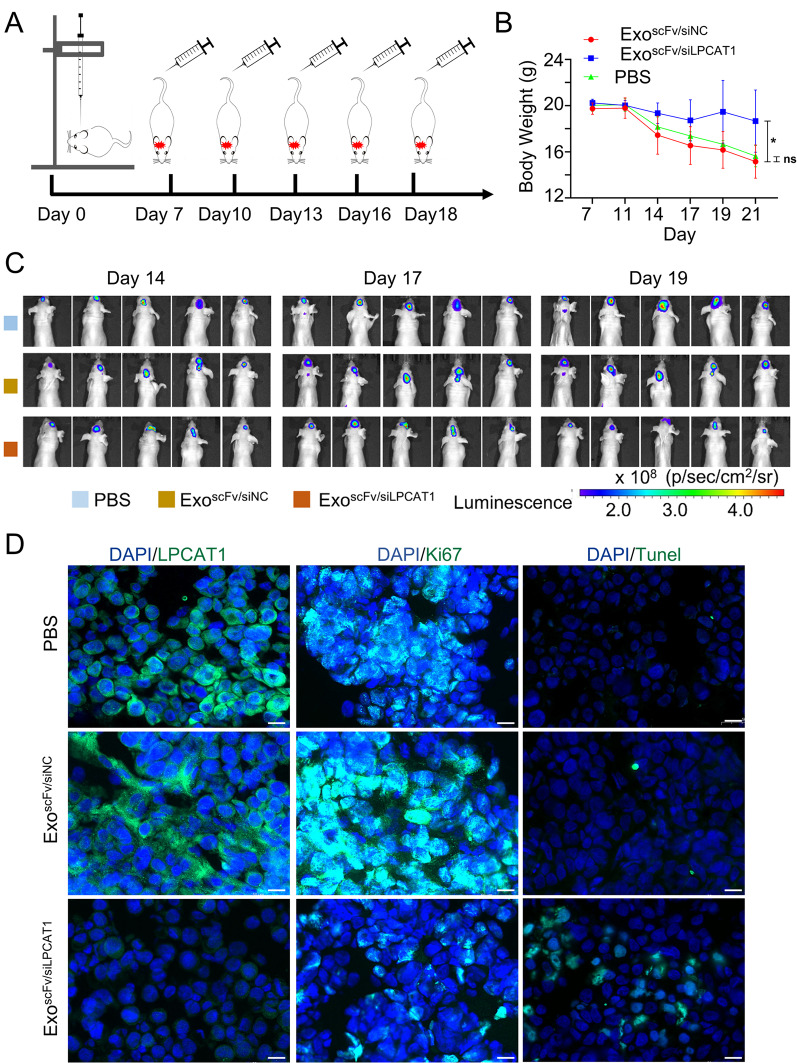
Anti-tumor effect of exoscFv/siLPCAT1 in vivo. (**A**) Schematic illumination of the experimental procedure. (**B**) Change of body weight of BALB/C nudemice treated with exoscFv/siLPCAT1, exoscFv/siNC or PBS, *p < 0.05, ns, not significance, n = 5 mice per group. Exosomes dosage = 5 mg/kg. (**C**) Representativebioluminescent images of BM tissues of nude mice treated with exoscFv/siLPCAT1, exoscFv/siNC or PBS at indicated time intervals after injection, n = 5 miceper group. (**D**) The signals of LPCAT1 (Green), ki67 (Green) and tunel (Green) in BMs sections were detected by confocal fluorescence imaging. Scale bar= 50 μm

The original article has been corrected.

