# Supplementary material for: Anti-EGFR ScFv functionalized exosomes delivering LPCAT1 specific siRNAs for inhibition of lung cancer brain metastases
Source: J Nanobiotechnology. 2024 Apr 8;22:159. doi: 10.1186/s12951-024-02414-7 (PMC11000333; doi:10.1186/s12951-024-02414-7)
Supplement: Supplementary file 1 — Supplementary Material 1 [file 12951_2024_2414_MOESM1_ESM.docx]

**Supplementary Information**

**Anti-EGFR ScFv Functionalized Exosomes Delivering LPCAT1 Specific siRNAs for** **Anti-EGFR ScFv Functionalized Exosomes Delivering LPCAT1 Specific siRNAs for Inhibition of Lung Cancer** **Brain Metastases**

Jun Jiang^a^**^,^**^1^, Yuan Lu^b,1, *^, Jie Chu^c,1^, Xiao Zhang^c^, Chao Xu^d^, Shaojie Liu^d^, Zhuo Wan^e^, Jiawei Wang^f^, Lu Zhang^c^, Kui Liu^a^, Zhenhua Liu^a^, Angang Yang^h^, Xinling Ren^g^, Rui Zhang^c,h*^

^a^ Department of Health Service, Base of Health Service, Air Force Medical University, Xi’an, China

^b^ Department of Respiratory and Critical Care Medicine, Zhongda Hospital, Southeast University, Nanjing, China

^c^ State Key Laboratory of Cancer Biology, Department of Biochemistry and Molecular Biology, Air Force Medical University, Xi’an, China

^d^ Department of Urology, Xijing Hospital, Air Force Medical University, Xi'an, China.

^e^ Department of Hematology, Tangdu Hospital, Air Force Medical University, Xi'an, China

^f^ Basic Medicine School, Air Force Medical University, Xi'an, China

^g^ Department of Respiratory and Critical Care Medicine, Shenzhen General Hospital, Shenzhen University, Shenzhen, China

^h^ State Key Laboratory of Cancer Biology, Department of Immunology, Air Force Medical University, Xi’an, China

^1^ Jun Jiang, Yuan Lu and Jie Chu contribute equally to this work.

* Corresponding author：

Yuan Lu, Department of Respiratory and Critical Care Medicine, Zhongda Hospital, Southeast University, Nanjing 210009, China, E-mail: lulu2023@126.com

Xinling Ren, Department of Respiratory and Critical Care Medicine, Shenzhen University General Hospital, Shenzhen University, Shenzhen, Guangdong 518055, China, E-mail: majrenxl@fmmu.edu.cn.

Rui Zhang, State Key Laboratory of Cancer Biology, Department of Immunology, Air Force Medical University, Xi’an, Shaanxi 710032, China, E-mail: ruizhang@fmmu.edu.cn.

**Content**

Fig S1. Stability of exo^scFv^.

Fig S2. Luminescence imaging of tumor and major organs from PC9 lung cancer tumor-bearing mice.

Fig S3. Luminescence imaging of PC9 lung cancer brain metastases mice model.

Fig S4. Encapsulation efficiency measurement of siRNA

Fig S5. Western blot analysis of EGFR and LPCAT1.

Fig S6. Quantitation of tumor luminescent intensity at 19^th^ day

Fig S7. Quantitation of TUNEL and Ki67 positive cells in tumors.

Table S1. The sequence of the scFv-lamp2b fusion protein

**
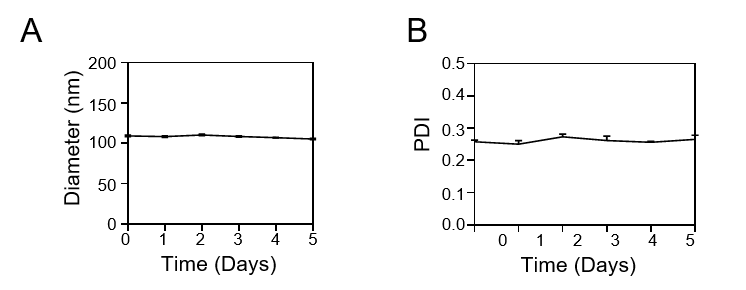
Fig S1. Stability of** **exo^scFv^.** Diameter (A) and polydispersity index (PDI) (B) of exo^scFv^ were measured by ZetaView Particle Metrix at different time periods after being incubated in DMEM medium containing 10% FBS. The data are shown as the means ± SEM of n = 3 independent samples.

**
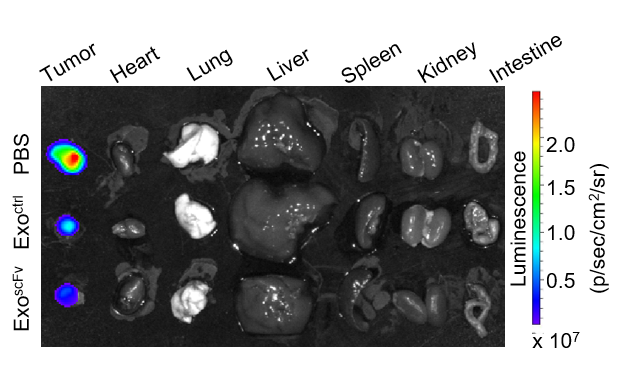
Fig S2. Luminescence imaging of tumor and major organs in PC9 tumor-bearing model.** The unilateral tumor model was established by subcutaneously inoculating 2 × 10^6^ luciferase expressing PC9 cells (PC9-Luc) into the right flank of BALB/C nude mice. When the lung cancer reached 50 mm^3^, tumor and major organs were collected and analyzed using IVIS Lumina III Living Image system after injecting 150 mg/kg luciferin.


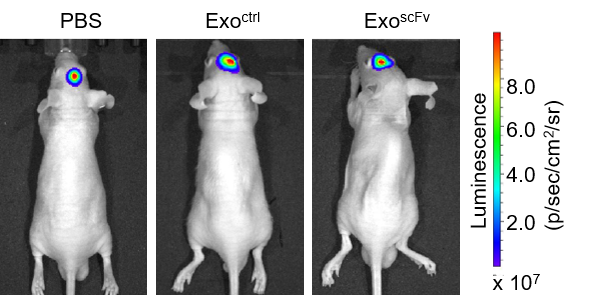
**Fig S3. Luminescence imaging of lung cancer** **brain metastases mice model.** The brain metastases model was established by intraparenchymally injecting 5×10^5^ luciferase expressing PC9 cells (PC9-Luc) into the striatum of BALB/c nude mice. Seven days later, tumor luminescence in mice brain was detected by IVIS Lumina III Living Image system after injecting 150 mg/kg luciferin.**
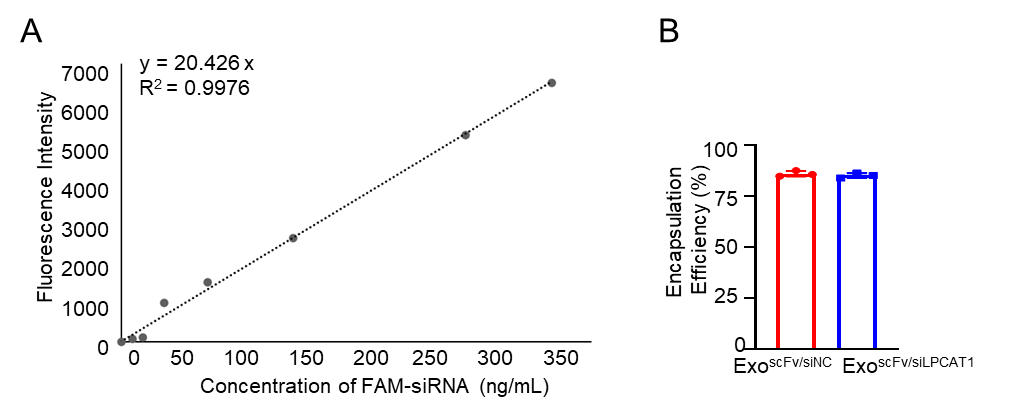
Fig S4. Encapsulation efficiency of siRNA. (A)** Standard curve of FAM-siRNA fluorescence intensity. (**B)** The encapsulation efficiency of siNC or siLPCAT1 in exo^scFv^. The data were shown as the means ± SEM of 3 independent samples.


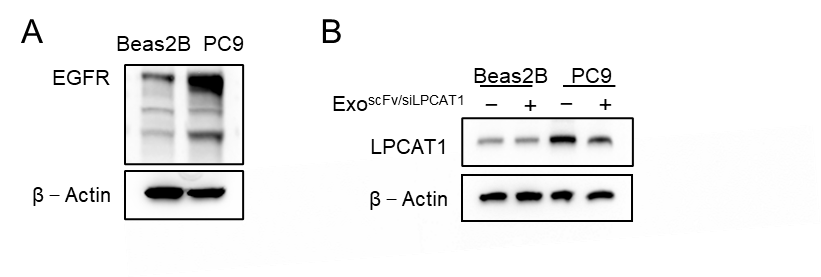


**Fig S5. Western blot analysis of EGFR and LPCAT1 expression in PC9 and Beas2b cells** with or without exo^scFv/siLPCAT1^ treatment**.** (A) EGFR expression in Beas2B and PC9 cells. (B) LPCAT1 expression in Beas2B and PC9 cells.


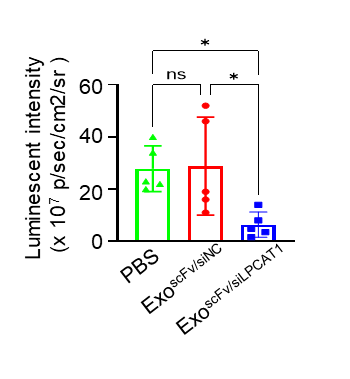


**Fig S6. Quantitation of tumor luminescent intensity in brain at the 19^th^ day**. n= 5 mice per group, **p* < 0.05, ns, not significance.

**
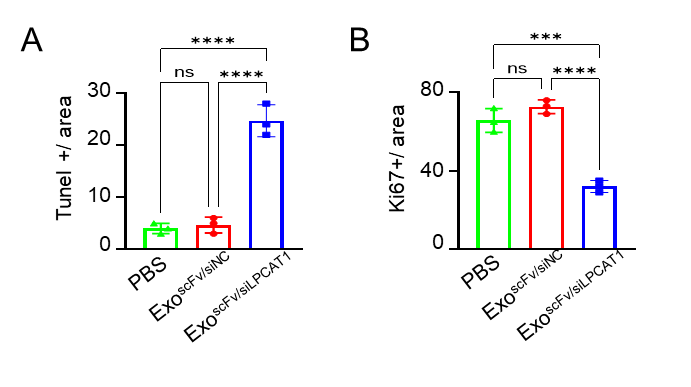
**

**Fig S7. Quantitation of TUNEL and Ki67 positive cells in tumors.** The data are shown as the means ± SEM, n = 3. ****p* < 0.001, *****p* < 0.0001; ns, not significance.

Table S1. The sequence of the scFv-lamp2b fusion protein.

| scFv | MVCFRLFPVPGSGLVLVCLVLGAVRSYALQQSGAEVKKPGSSVKVSCKASGYTFTNNYIYWVRQAPGQGLEWIGGINPTSGGSNFNEKFKTRVTITADESSTTAYMELSSLRSEDTAFYFCTRQGLWFDSDGRGFDFWGQGTTVTVSGGGGSGGGGSGGGGSIQMTQSPSSLSASVGDRVTITCRSSQNIVHSNGNTYLDWYQQTPGKAPKLLIYKVSNRFSGVPSRFSGSGSGTDFTFTISSLQPEDIATYYCFQYSHVPWTFGQGTKLQILELNLTDSENATCLYAKWQMNFTVRYETTNKTYKTVTISDHGTVTYNGSICGDDQNGPKIAVQFGPGFSWIANFTKAASTYSIDSVSFSYNTGDNTTFPDAEDKGILTVDELLAIRIPLNDLFRCNSLSTLEKNDVVQHYWDVLVQAFVQNGTVSTNEFLCDKDKTSTVAPTIHTTVPSPTTTPTPKEKPEAGTYSVNNGNDTCLLATMGLQLNITQDKVASVININPNTTHSTGSCRSHTALLRLNSSTIKYLDFVFAVKNENRFYLKEVNISMYLVNGSVFSIANNNLSYWDAPLGSSYMCNKEQTVSVSGAFQINTFDLRVQPFNVTQGKYSTAQECSLDDDTILIPIIVGAGLSGLIIVIVIAYVIGRRKSYAGYQTLHHHHHH |
| --- | --- |
