# Supplementary material for: Anti-EGFR ScFv functionalized exosomes delivering LPCAT1 specific siRNAs for inhibition of lung cancer brain metastases
Source: J Nanobiotechnology. 2024 Apr 8;22:159. doi: 10.1186/s12951-024-02414-7 (PMC11000333; doi:10.1186/s12951-024-02414-7)
Supplement: Supplementary file 3 — Supplementary Material 3 [file 12951_2024_2414_MOESM3_ESM.docx]

**Anti-EGFR ScFv Functionalized Exosomes Delivering LPCAT1 Specific siRNAs for Inhibition of Lung Cancer** **Brain Metastases**

Jun Jiang^a^**^,^**^1^, Yuan Lu^b,1, *^, Jie Chu^c,1^, Xiao Zhang^c^, Chao Xu^d^, Shaojie Liu^d^, Zhuo Wan^e^, Jiawei Wang^f^, Lu Zhang^c^, Kui Liu^a^, Zhenhua Liu^a^, Angang Yang^h^, Xinling Ren^g^, Rui Zhang^c,h*^

^a^ Department of Health Service, Base of Health Service, Air Force Medical University, Xi’an, China

^b^ Department of Respiratory and Critical Care Medicine, Zhongda Hospital, Southeast University, Nanjing, China

^c^ State Key Laboratory of Cancer Biology, Department of Biochemistry and Molecular Biology, Air Force Medical University, Xi’an, China

^d^ Department of Urology, Xijing Hospital, Air Force Medical University, Xi'an, China.

^e^ Department of Hematology, Tangdu Hospital, Air Force Medical University, Xi'an, China

^f^ Basic Medicine School, Air Force Medical University, Xi'an, China

^g^ Department of Respiratory and Critical Care Medicine, Shenzhen General Hospital, Shenzhen University, Shenzhen, China

^h^ State Key Laboratory of Cancer Biology, Department of Immunology, Air Force Medical University, Xi’an, China

^1^ Jun Jiang, Yuan Lu and Jie Chu contribute equally to this work.

* Corresponding author：

Yuan Lu, Department of Respiratory and Critical Care Medicine, Zhongda Hospital, Southeast University, Nanjing 210009, China, E-mail: lulu2023@126.com

Xinling Ren, Department of Respiratory and Critical Care Medicine, Shenzhen University General Hospital, Shenzhen University, Shenzhen, Guangdong 518055, China, E-mail: majrenxl@fmmu.edu.cn.

Rui Zhang, State Key Laboratory of Cancer Biology, Department of Immunology, Air Force Medical University, Xi’an, Shaanxi 710032, China, E-mail: ruizhang@fmmu.edu.cn.

**Abstract**

Brain metastasis (BM) is one of the leading causes of cancer-related deaths in patients with advanced non-small cell lung cancer (NSCLC). However, limited treatments are available due to the presence of the blood-brain barrier (BBB). Upregulation of lysophosphatidylcholine acyltransferase 1 (LPCAT1) in NSCLC has been found to promote BM. Conversely, downregulating LPCAT1 significantly suppresses the proliferation and metastasis of lung cancer cells. In this study, we firstly confirmed significant upregulation of LPCAT1 in BM sites compared to primary lung cancer by analyzing scRNA dataset. We then designed a delivery system based on a single-chain variable fragment (scFv) targeting the epidermal growth factor receptor (EGFR) and exosomes derived from HEK293T cells to enhance cell-targeting capabilities and increase permeability. Next, we loaded LPCAT1 siRNA (siLPCAT1) into these engineered exosomes (exo^scFv^). This novel scFv-mounted exosome successfully crossed the BBB in an animal model and delivered siLPCAT1 to the BM site. Silencing LPCAT1 efficiently arrested tumor growth and inhibited malignant progression of BM *in vivo* without detectable toxicity. Overall, we provided a potential platform based on exosomes for RNA interference (RNAi) therapy in lung cancer BM.

**Keywords**: Lung cancer，Engineered exosomes，Drug delivery, Brain metastasis，Blood-brain barrier

**Introduction**

Lung cancer is the leading cause of cancer-related deaths worldwide, resulting in approximately 350 deaths per day [[1](#_ENREF_1)]. Brain metastases (BM) are the main cause of high mortality in lung cancer patients [[2](#_ENREF_2)]. Lung cancer is also the most common primary tumor that metastasizes to the brain, accounting for 40%-50% of cases, which is significantly higher than breast cancer (15%-25%) and melanoma (5%-20%) [[3](#_ENREF_3), [4](#_ENREF_4)]. While tyrosine kinase inhibitors (TKIs) targeting mutant epidermal growth factor receptor (EGFR) have improved the survival of patients with non-small cell lung cancer (NSCLC) in the last decade, NSCLC with mutated EGFR are more likely to spread to the brain than those with wild-type EGFR [[5-7](#_ENREF_5)]. Despite the promising initial responses to TKIs, patients eventually develop acquired drug resistance [[8](#_ENREF_8)]. As a result, patients often experience central nervous system (CNS) relapse and succumb to the disease. Although immune checkpoint inhibitors targeting the PD-1-PD-L1 axis have achieved success as a standard treatment for a variety of cancers, the occurrence of immune-related toxicities and tolerance in a significant number of patients limits its effectiveness in treating BM (only 17-44% of BM patients benefit from this treatment) [[9](#_ENREF_9)]. Therefore, there is an urgent need to develop more effective therapeutic strategies to suppress lung cancer brain metastases. Nevertheless, one major obstacle to delivering effective therapeutics to the tumor sites is the blood-brain barrier (BBB).

Exosomes are nano-scale (30-200nm) extracellular vesicles that can be secreted into the bloodstream by various types of cells, exerting biological functions remotely. Exosomes play an important role in tumorigenesis, cancer metastasis, and drug resistance by remodeling the tumor microenvironment through the transfer of exosomal contents to recipient cells [[10](#_ENREF_10)]. Exosomes have been shown to be a natural carrier with low immunogenicity and high compatibility, making them an ideal tool for transporting therapeutic reagents for cancer treatment. For example, in a mouse model of brain metastases of breast cancer, CXCR4/TRAIL-enriched exosomes enhanced the anti-tumor efficacy of chemotherapy, while exosomes-based delivery of cPLA2 siRNA and metformin targeting intracranial xenografts of glioblastoma suppressed the energy metabolism of tumor cells and exerted cell growth inhibitory effects [[11](#_ENREF_11), [12](#_ENREF_12)]. However, the tumor treatment based on the delivery of exosomes is not sufficiently accurate or efficient. Therefore, targeting antibodies or peptides are often anchored on the exosome surface to introduce a cell-targeting feature and increase permeability [[13](#_ENREF_13)].

Lysophosphatidylcholine acyltransferase 1 (LPCAT1), a cytosolic enzyme that converts lysophosphatidylcholine into phosphatidylcholine, is highly expressed in multiple cancer types, including lung cancer [[14](#_ENREF_14)], glioblastoma [[15](#_ENREF_15)], endometrial cancer [[16](#_ENREF_16)], and esophageal carcinoma [[17](#_ENREF_17)]. LPCAT1 has been shown to significantly promote brain metastases of lung cancer by upregulating the PI3K/AKT/MYC pathway, making it a promising target for treating BM in lung cancer patients. Gene silencing of LPCAT1 remarkably reduced tumor cell proliferation *in vitro* and attenuated BM *in vivo* [[14](#_ENREF_14)].

Previously, we developed an EGFR-specific single-chain antibody fragment (scFv), which was used as a cell-targeting tool to deliver siRNA to EGFR-positive xenografts in a mouse model and to reverse drug resistance of EGFR-TKI [[18](#_ENREF_18)]. We also synthesized targeting nanoparticles based on this scFv and demonstrated its feasibility as a contrast agent for MRI *in vivo* [[19](#_ENREF_19)]. In this study, we designed a fusion protein consisting of scFv and exosomal surface protein lamp2b, which was expressed on the surface of exosomes derived from HEK293 cells to enable cell targeting and therapeutic functions. We then encapsulated the engineered exosomes armed to EGFR with LPCAT1 siRNA (siLPCAT1) and intravenously injected them into a mouse model of lung cancer BM (Fig.1). The engineered exosomes (exo^scFv^) displayed high intracranial permeability and delivery efficiency to the brain tumor. Importantly, our data showed that LPCAT1 siRNA-loaded scFv-coated exosomes (exo^scFv/siLPCAT1^) exerted potent anti-tumor effects in metastatic brain tumors from lung cancer.

**Materials and Methods**

**Data Retrieval and Preprocessing**

We employed a single-cell RNA sequencing (scRNA-Seq)-based profiling method to quantitatively determine the cell types and states within NSCLC. We firstly searched in the Gene Expression Omnibus (GEO) and finally included one cohort (GSE131907, Ahn M et al. [[20](#_ENREF_20)]) with scRNA-seq data of 58 tissue samples from 44 patients, consisting of 11 normal lung tissues, 11 primary tumor tissues, 10 brain metastases and other tissues. The clinical information of the GSE131907 dataset were available on the GEO database (https://www.ncbi.nlm.nih.gov/geo/). The scRNA-Seq data passed the quality control were then input into the Seurat R package (package v4.0.1) to obtain the unsupervised clustering based on the first 20 principal components of the top 2000 most variable genes among the whole-genome. UMAP (“RunUMAP”function) was used for the visualization of clustering [[21](#_ENREF_21)].

**Plasmid Construction**

Genes encoding the lamp2b and anti-EGFR scFv-lamp2b fusion protein were synthesized by AuGCT Technologies, Inc (Beijing, China). The scFv-lamp2b fusion protein, in which the scFv and lamp2b are linked via a flexible (GGGGS)3 peptide, contained a 6 × His.tag at the C-termini of lamp2b. To generate expression constructs of exo^scFv^, scFv gene fragments were cloned into the pcDNA3.1(-) vector between NheI and XhoI restriction enzyme sites. The generated expression constructs were confirmed by DNA sequencing (LC-Bio, Inc, Hangzhou, Zhejiang, China).

**Cell Culture and Preparation**

HEK293T cells and PC9 cells were purchased from Shanghai Institutes for Biological Sciences (Chinese Academy of Sciences, Shanghai, China). HEK293T cell lines are commonly used to express exogenous genes. Thus, scFv-modified exosomes derived from HEK293T cells are feasible vesicles for delivering therapeutic molecules [[22](#_ENREF_22)]. HEK293T cells were cultured in Dulbecco’s modified Eagle’s medium (DMEM; Gibco BRL, USA) with 10% fetal bovine serum (FBS, Biological Industries, Beit Ha’emek, Israel), 100 U/mL of penicillin, and 100 U/mL of streptomycin. Human PC9 cells were cultured in 1640 medium (Gibco, USA) with 10% FBS and 1% penicillin-streptomycin. All cells were incubated at 37°C in a 5% CO2 atmosphere. A luciferase plasmid was constructed based on the pLenti 6.3 lentiviral vector, and the methods of establishing PC9 clones stably expressing luciferase were similar to those described before [[23](#_ENREF_23)].

**Exosomes Production, Isolation, Characterization and half-life**

To generate exosomes that highly express surface anti-EGFR scFv-lamp2b (exo^scFv^) and lamp2b (exo^ctrl^), HEK293T cells were transfected with the expression constructs using transfection reagent lipofectamine 2000 (Invitrogen, Carlsbad, CA, USA). Six hours later, the cell culture medium was replaced with exosome-free medium and incubated for another 48 hours. The cell culture supernatants were then collected and centrifuged at 500 g for 10 min, 2,000 g for 10 min, and 10,000 g for 30 min, respectively, to remove cells, residual cell debris, and larger extracellular vesicles in sequence. After being filtered by 0.22 μm filters (Millipore), the supernatants were centrifuged at 140,000 g for 2 hours using an SW32 TI rotor in an Optima XE-100 ultracentrifuge (Beckman Coulter). The isolated exosome precipitates were re-suspended in EV-guardTM storage buffer (system biosciences, SBI, US, cat# EXSBA-1) and stored at −80°C. The exosomes were negatively stained by 4% uranyl acetate and observed using a transmission electron microscopy (TEM: JEM-1230, JEOL Ltd., Tokyo, Japan). The particle size distribution of the exosomes was measured by the nanoparticle tracking analysis (NTA) using a ZetaView Particle Metrix. Half-life (τ1/2) was evaluated using equation, τ1/2 = ln2/k = (t2－t1)*ln2/(lnC1－lnC2), (C1, fluorescence intensity at time 1 (t1); C2, fluorescence intensity at time 2 (t2)).

**Western Blot Analysis**

To validate whether the fusion protein was successfully expressed in 293T cells and integrated into the exosome membranes, we performed western blot using anti-His antibody. Total protein of isolated exosomes or cells was extracted in RIPA Lysis Buffer (Solarbio, China) at 4°C for 15 min. The protein concentration was determined using a BCA protein assay kit (Solarbio, China). About 30 μg of protein was separated on 10% SDS-PAGE gels, and then transferred to nitrocellulose membranes (Millipore). The membranes were blocked with 3% BSA, and then incubated with Anti-His-tag (1:5000, Abmart, China, #M20001), and anti-β-actin (1:4000, Sigma, St Louis, MO, USA) at 4 °C overnight. Anti-CD63 (1:2000, Proteintech, Wuhan, Hubei, China, #25682-1-AP), anti-GM130 (1:2000, Proteintech, #11308-1-AP), anti-TSG101 (1:2000, Proteintech, #28283-1-AP) were also used to characterize the exosomes. anti-LPCAT1 (1:2000, Proteintech, #16112-1-AP), anti-EGFR (1:5000, Proteintech, #18986-1-AP). This procedure was followed by adding HRP-conjugated secondary antibody (1:1000, Cell Signaling Technology, Beverly, MA, USA) and ECL reagents (Solarbio, Beijing, China). The bands were visualized and recorded using the Tanon 5500 imaging system.

**Exosomes Labeling and Targeting in vitro**

For *in vitro* experiments, the PKH76 fluorescent dye (Umibio, Shanghai, China) was used to label exosomes according to the manufacturer's instructions. PC9 Cells were seeded in confocal dishes, and co-incubated with PKH67-labeled exo^scFv^/exo^ctrl^ for 3h and 6h. The cells were then fixed in 4% (w/v) paraformaldehyde in PBS for 15 min, and the nuclei were stained with 1.5 µg/mL of 4,6-diamidino-2-phenylindole dihydrochloride (DAPI, Sigma-Aldrich, USA) at room temperature for 5 min. After washing in PBS, the intracellular distribution of exosomes was visualized with a fluorescence microscope (Nikon, Tokyo, Japan).

**Preparation of siRNA-Loaded Exosomes and Encapsulation Efficiency**

Protein concentration of exosomes was measured using a BCA assay. Exo^scFv^/exo^ctrl^ (100 µg with a protein concentration of 1 µg/µL) was mixed with 5 OD of siRNA at 4°C for 30 min (The total volume was no more than 400 µL). The mixtures were then added into electroporation cuvettes (cap size: 2 mm). Electroporation was performed using the Gene Pulser Xcell Electroporation System (Bio-Rad, Hercules, CA, USA) and the electroporation condition was as followed: 350 V, 150 mA, 2 pulses. After electroporation, the exosomes were cooled on ice for 30 min.

To calculate the encapsulation efficiency of siRNA, various concentration of FAM labeled siRNA was diluted in DEPC water. A fluorescence standard curve was plotted based on different concentrations (0, 8, 16, 32, 64, 128, 256 and 320 ng/ml) of FAM-siRNA. After electroporation, the exosomes were centrifuged and resuspended in fresh PBS. The fluorescence intensity of free FAM-siRNA in the supernatant was detected and the concentration of free FAM-siRNA (C_free siRNA_, ng/ml) was calculated. Encapsulation Efficiency (EE%) = (W_siRNA_ - C_free siRNA_ × V_free siRNA_) / W_siRNA_ ×100%. W_siRNA_, total weight of siRNA, V_free siRNA_, volume of siRNA.

**Quantitative Real‑Time PCR**

Total RNA was extracted from cells using TRIzol reagent (Invitrogen, Carlsbad, CA, USA). cDNA was synthesized using a Prime Script qRT-PCR Kit (Takara Bio Inc., Japan). The expression level of LPCAT1 was tested by qPCR (Bio-Rad CFX Manager 3.1). Correlation data were calculated based on the ΔΔCT of the target gene and the internal reference actin. The primer sequences used were as follows (F, forward; R, reverse): LPCAT1-F: ACATCCCGATCTGGGGAACT; LPCAT1-R: GGCCACTTTCCGTTGGACT. actin-F: CCTGGGCATGGAGTCCTGTG; actin-R: TCTTCATTGTGCTGGGTGCC.

**Cell apoptosis and proliferation assay**

The effect of different exosome formulations on cell apoptosis of PC9 was assessed by using the AnnexinV-FITC/PI kit (Bestbio, Shanghai, China). Cell proliferation-associated ki67 were evaluated by FCM assay using APC antihuman Ki-67 antibody (Biolegend, San Diego, USA). Flow cytometry data were analyzed by Flow Jo software. Cell Counting Kit-8 (CCK-8) assay was also performed to determine the effect of exosomes on cell proliferation. All procedures were according to the manufacturer’s instruction.

**Animal experiments**

All experimental procedures involving animals were conducted under a protocol reviewed and approved by the Ethics Committee of Air Force Medical University. 6-week-old of male BALB/C nude mice (18-20 g) were purchased from Vital River Laboratory Animal Technology Co., Ltd, Beijing, China. To evaluate the distribution of scFv modified exosomes, we firstly establish lung cancer tumor-bearing mice models, 2×10^6^ luciferase expressing PC9 cells were injected subcutaneously in the thigh of BALB/c nude mice. Approximately one week later, the tumor masses were visible to the naked eye. Nude mice were randomly assigned to three groups (PBS group, exo^ctrl^ group, exo^scFv^ group, n = 3). Every 200 µg exosomes (1 µg/µL) were then stained with 200µl 1,1'-Dioctadecyl-3,3,3',3'-Tetramethylindodicarbocyanine, 4-Chlorobenzenesulfonate Salt (Did, Beyotime, Shanghai, China) (20 µM) at 37°C for 10 min, followed by centrifugal isolation as described above. 200 µg DiD-labelled exo^scFv^/exo^ctrl^ (1 µg/µL) were injected via the tail vein at a dosage of 10 mg/kg. The distribution of the exosomes in different organs were then analyzed by the IVIS imaging system (PerkinElmer, life sciences, USA). Identical illumination settings were used as follows: ex: 620 nm/em: 670 nm, Filter Position 5, 10 cm feld of view, Binning factor of 4, 2 sec exposure time.

Lung cancer BM model was established by intraparenchymally injecting 5×10^5^ luciferase expressing PC9 cells into striatum of BALB/c nude mice [[24](#_ENREF_24)]. Briefly, the mice were anesthetized and placed into a stereotactic apparatus (RWD, Shenzhen, China). A small hole was drilled 2 mm lateral and 1 mm anterior to the bregma. PC9 cells were then injected into the right hemisphere at a depth of 3 mm using a 10 µl Hamilton syringe with a 30-gauge needle. The injection was conducted at a constant speed for over 2 minutes followed by an additional 2 minutes pause before removing the needle. Then the scalp was stitched. One week later, the tumor in mice brain were observed after injection of 150 mg/kg Dluciferin. Nude mice were randomly assigned to three groups (PBS group, exo^ctrl^ group, exo^scFv^ group, n = 3). 200 µg DiD-labelled exo^scFv^/exo^ctrl^ (1 µg/µL) were injected via the tail vein as well, at a dosage of 10 mg/kg. Penetration of exosomes was assessed by the IVIS imaging system.

To evaluate anti-tumor effects, mice with BM tumor were randomly divided into three groups (n= 4) and injected with siLPCAT1-loaded exo^scFv^/exo^ctrl^ (exo^scFv/siLPCAT1^/exo^scFv/siNC^, 1 µg/µL) or PBS via the tail vein every 3 day for a total 4 times, at a dosage of 5 mg/kg. The tumor location and volume in brain was monitored by detecting bioluminesence after injecting 150 mg/kg luciferin. Identical illumination settings were used as follows:ex: block/em: open, 10 cm feld of view, Binning factor of 4, 0.5 sec exposure time.

**Immunofluorescence assay**

Mice brains were fixed in 4% paraformaldehyde for 15 min and carefully frozen in OCT freezing media then dissected into 8 µm thick slices. Cell nuclei were stained by DAPI (Invitrogen). The fluorescence of DiD-labelled exosomes was observed by EVOS FL Auto 1 Cell Imaging System. To evaluate the expression of LPCAT1 and Ki67 in mice brains, sections were incubated with the respective primary antibodies (Anti-LPCAT1 (Proteintech), anti-Ki67 (Cell Signaling Technology, Danvers, MA, USA)) at room temperature for 4h. To detect apoptotic tumor cells within brain metastases after treatment, sections were stained with *in situ* Tunel kit (Roche, Mannheim, Germany).

**In vivo safety evaluation**

Ten male BALB/c nude mice were randomly divided into two groups. One group receivedintravenous injection of 20 mg/kg exo^scFv/siLPCAT1^every other day for one week and the other group was treated with PBS as control. Mice were then sacrificed to harvest the tissues and blood samples at 24 h after the last administration. The whole blood samples were stored at room temperature for 2h and centrifuged (4,000 g) at 4°C for 10 min. The serum biochemical parameters including liver enzymes, and renal function were measure by automatic biochemical analyzers (Chemray 800 and Chemray 240, Rayto, China). Major organs, including heart, liver, spleen, lung and kidney, were fixed with 4% paraformaldehyde, embedded with paraffin, and then stained with hematoxylin-eosin (H&E). Tissue sections were also investigated for LPCAT1 expression by immunohistochemistry (IHC) to evaluate whether the exo^scFv/siLPCAT1^ alter LPCAT1 expression in major organs.

**Statistical analysis**

All Data are presented as the mean ± standard deviation. All statistical analyses were conducted with GraphPad Prism 8.0. The statistical significance values between two groups were determined by the *Student* *t* test. One-way ANOVA was used for comparing the differences between groups. Probability values < 0.05 were considered statistical significance.

**Results**

**LPCAT1 was highly upregulated in malignant cells of lung cancer brain metastases**

LPCAT1 was supposed to play a pro-tumoral role in NSCLC by promoting lung cancer cell proliferation and migration [[14](#_ENREF_14)]. To further understand the heterogeneity and meaning of LPCAT1 expression in NSCLC primary tumors and metastatic lesions, we obtained and analyzed the lung adenocarcinoma brain metastases single cell transcriptome sequencing dataset (GSE131907). After data processing and annotation, uniform manifold approximation and projection (UMAP) profiles and 27 cellular subtypes were stratified. The 30,272 epithelial cancer cells were cataloged into 3 clusters with 0.8 resolution that primary lung cancer cells, metastatic brain tumor cells and metastatic lymph node cancer cells could optimally be separated by UMAP plot, suggesting significant heterogeneity in malignant cells between brain metastases and primary tumor (Fig.2A-B). Moreover, the expression of LPCAT1 was found to be significantly elevated, specifically in BM samples, suggesting malignant cancer cells with upregulated LPCAT1 were prone to migrate to the brain (Fig.2C-D).

**Isolation and characterization of anti-EGFR scFv-expressed exosomes**

To confer targeting capability of exo^scFv^, we fused the anti-EGFR scFv to the exosomal membrane protein lamp2b, a protein abundantly expressed on exosomal membranes [[25](#_ENREF_25)]. The coding sequence of anti-EGFR scFv was inserted into the fusion protein between the signal peptide and mature peptide coding sequence. A 6×his.tag was attached to the C terminus of lamp2b to confirm the expression of the recombinant protein and enable the location of exo^scFv^. A fusion protein containing merely lamp2b and his.tag was also constructed as the control exosomes referred to as exo^ctrl^ (Fig. 3A). The coding sequence was cloned into pcDNA3.1 (-) plasmids and then transfected into HEK293T cells (Fig. 3B). The exosomes were isolated and purified from the culture supernatants of 293T cells by ultracentrifugation. As shown in Fig. 3C, scFv-lamp2b was successfully expressed, and lamp2b was upregulated in the corresponding 293T cell and derived exosomes. The molecular weight of scFv-lamp2b-his.tag and lamp2b-his.tag fusion proteins was around 100 kd and 80 kd, respectively. The presence of exosomal markers CD63 and TSG101 was also confirmed by WB assay, while the Golgi apparatus marker GM130 was barely observed. The physical properties of modified exosomes were analyzed by TEM and NTA analysis. TEM was used to directly visualize the exosomes. Both exo^ctrl^ and exo^scFv^ had typical displayed saucer-like bilayer morphologies (Fig. 3D). NTA measurements showed that both kinds of exosomes had homogeneous particle size distribution with a mean diameter of 109.4 nm and 110.0 nm respectively, ranging from 30-150 nm in diameter. (Fig. 3E). In the stability test (Fig. S1), the size and polydispersity index (PDI) of incubation of exo^scFv^ remained at approximately 110nm and 0.25, respectively, after five days of incubation in medium containing 10% FBS, indicating that exo^scFv^ could remain stable in the physiological environment.

**In vitro targeting of exo^scFv^**

To evaluate the potential tumor targeting ability of exo^scFv^ in vitro, we labeled the exosomal part of exo^scFv^ and exo^ctrl^ with PKH76. As shown in Fig. 3F, a clear cytoplasmic fluorescent signal of PKH76 (green) in EGFR positive lung cancer PC9 cells was observed, which represents the uptake of exo^scFv^ and exo^ctrl^. A higher signal intensity was visible after 3h of incubation in the exo^scFv^ group compared to the exo^ctrl^ group. The intensity difference between the two groups became more pronounced after 6h of incubation, suggesting that the anti-EGFR scFv anchored on the extra-exosomal membrane enhanced the ability to recognize antigens on lung cancer cells and could rapidly guide exosomes to receiving cells and accelerate its internalization.

**In vivo distribution of exo^scFv^**

To assess the tumor-targeting ability of exo^scFv^, we firstly established the tumor-bearing mice models, 200 μg DiD-labeled exo^ctrl^ or exo^scFv^ was injected via tail vein (Fig.4A). The biodistribution of exosomes was monitored at 1h, 24h, 48h, 96h using IVIS. As shown in Fig.4B, sufficient fluorescence could be yielded since 1h post-injection and gradually diffused throughout the whole bodies, especially reticuloendothelial systems, such as liver, lung, and spleen. However, the fluorescence intensity in tumor masses of the control group began to weaken before 48h after injection (Fig.4C). In contrast, the tumor fluorescence intensity of the exo^scFv^ group reached the peak at 48h. Moreover, the residual fluorescence intensity of the exo^scFv^ group at 96h was two times higher than that of the exo^ctrl^ group. Notably, there were abundant exosomes detained in tumor verified by *Ex* vivo fluorescence imaging (Fig.4C and Fig.S2). In addition, fluorescence imaging of frozen tissue sections showed DiD-labeled exosomes were detected mainly in the lung and liver, followed by the kidney, heart, and tumor, consistent with the results of *in vivo* imaging (Fig.4E). Additionally, the half-life of exo^scFv^ in lung, kidney, liver and tumor is 14.51 h, 22.23 h, 32.89 h and 71.37 h, respectively. These results suggested that EGFR-scFv modification effectively enhanced the tumor targeting of exosomes and prolonged the retention time of exosomes in the tumor.

To further evaluate the brain permeability and BM lesions targeting ability of exo^scFv^, we developed the animal BM model according to the previous method [[26](#_ENREF_26)]. PC9 cells stably expressing luciferase were injected into the striatum of *BALB/c* nude mice by intraparenchymal implantation. Tumor growth was monitored by bioluminescence imaging. One week later, *in vivo* imaging showed that the BM model was successfully established as IVIS-positive foci were observed at the implantation sites (Fig. S3). The tumor-bearing mice were injected with DiD-labeled exo^ctrl^ or exo^scFv^ via tail vein, respectively. The biodistribution of exosomes was monitored at 0.5h, 3h, 6h, 24h using IVIS. 24-hour IVIS imaging was used in this study. As shown in Fig. 5A-B, imaging at 3 hours after injection revealed a rapid accumulation of exo^scFv^ in the tumor site of mice brain, as the fluorescence intensity was much greater than that of the exo^ctrl^ group. Notably, there were abundant exosomes localized in the brain (Fig. 5C-D). Additionally, as shown in Fig. 5E-F, PC9 BM slices from the brain of the mice injected with exo^scFv^ exhibited significantly increased DiD signal intensity than those from the mice injected with exo^ctrl^, and no signal was observed in adjacent normal brain tissues. Importantly, the fluorescence difference of the two groups appeared earlier in the BM model, which might be due to the presence of the BBB, as exosomes have to enter the tumor site at a slower pace.

**Anti-tumor effect of exo^scFv/siLPCAT1^ in vitro**

To verify whether LPCAT1 could become a therapeutic target for NSCLC BM treatment, in this study, we firstly prepared exo^scFv^ loading with siLPCAT1. Under the optimum electroporation conditions, the encapsulation efficiency of exo^scFv^ for siLPCAT1 and siNC were 85.19 ± 1.21 % and 86.07 ± 1.39 %, respectively (Fig. S4). Then we evaluated the gene silencing effectiveness of LPCAT1 siRNA loaded exosomes (exo^scFv/siLPCAT1^) in PC9 cells. PC9 cells has much higher expression of EGFR than Beas2b lung epithelial cell (Fig. S5A). And exo^scFv/siLPCAT1^ resulted in a marked decrease expression of LPCAT1 in PC9 compared to the control (Fig. 6A-B, Fig.S5B). Next, we explored the role of exo^scFv/siLPCAT1^ in lung cell cancer proliferation and apoptosis by performing CCK8 and FCM assays. The CCK8 assay revealed that cell proliferation was significantly repressed in the exo^scFv/siLPCAT1^ group compared with the control (Fig. 6C). Similarly, the FCM assay showed that the proliferative activity of PC9 cells was significantly decreased in the exo^scFv/siLPCAT1^ group (Fig. 6D-E). The FCM assay also revealed that exo^scFv/siLPCAT1^-treated PC9 cells had a significantly higher late apoptotic rate (13.03 ± 0.73%) than exo^scFv/siNC^-treated cells (5.84 ± 0.88%) (p < 0.001), and only 7.86 ± 1.13% exo^scFv^ (p < 0.001) and 5.86 ± 1.27% PBS-treated cells (p < 0.001) were undergoing apoptosis (Fig. 6G-F).

**In vivo anti-tumor effect of exo^scFv/siLPCAT1^**

We next explored whether EGFR specific scFv functionalized exo^scFv^ could efficiently deliver siLPCAT1 across BBB to the BM site of lung cancer *in vivo*. The schematic representation of the *in vivo* assay procedure was shown in Fig. 7A. The body weight of mice was recorded the day after injection, and the tumor growth was monitored by IVIS during the same period. As shown in Fig. 7B, mice in the exo^scFv/siLPCAT1^ treated group lost less body weight by the first week than the PBS or exo^scFv/siNC^-treated mice, and body weight loss became significant from the 12^th^ day until the 14^th^ day. We also visually testified that exo^scFv/siLPCAT1^ had a superior anti-tumor effect compared with the control group, as the average load of brain metastases measured photon flux in IVIS was significantly lower. The tumor in the mice treated with PBS or exo^scFv/siNC^ grew rapidly and emitted strong luminescence. The exo^scFv/siLPCAT1^ treated group displayed a significantly smaller area and weaker intensity of bioluminescence, and the quantitation revealed that tumor luminescent intensity at 19^th^ day was approximately 4.51-folds lower in exo^scFv/siLPCAT^ than in the exo^scFv/siNC^ group (Fig. 7C, Fig. S6). To testify the silencing efficacy of siLPCAT1 *in vivo*, we further detected the expression of LPCAT1 in the BM site by immunofluorescence staining analysis. The expression of LPCAT1 in tumors from the exo^scFv/siLPCAT1^ group was significantly downregulated compared with those from the PBS and exo^scFv/siNC^ group (Fig. 7D). Meanwhile, to elucidate the cellular proliferation and apoptosis alteration of BM under the impact of LPCAT1 downregulation, the TUNEL and Ki67 staining were performed. As shown in Fig. 7D and Fig. S7A, the number of TUNEL positive cells was significantly increased in tumors from mice treated with exo^scFv/siLPCAT1^ compared with those from mice treated with PBS or exo^scFv/siNC^. Correspondingly, a significant decrease in the number of Ki67-positive cells was also detected in tumors from the exo^scFv/siLPCAT1^ group (Fig. 7D and Fig. S7B).

**Safety evaluation of exo^scFv/siLPCAT1^**

We investigated the toxicity of exo^scFv/siLPCAT1^ on mice and collected serum samples at a dosage of 20 mg/kg every other day for one week to measure aspartate aminotransferase (AST), alanine aminotransferase (ALT), blood urea nitrogen (BUN)，and creatinine (CRE). As shown in Fig.8A, no obvious hepatic or renal toxicity was observed in the exo^scFv/siLPCAT1^ treated group compared to the PBS treated group. Additionally, we examined hematoxylin and eosin (H&E)-stained sections of major organs, including the heart, lung, liver, spleen, and kidney from the exo^scFv/siLPCAT1^ and PBS treated groups. No evidence of structural damage was observed in the exo^scFv/siLPCAT1^ group as well (Fig. 8B). Meanwhile, there was no significant difference between the two groups in the expression of LPCAT1 in the tissues of the lung, liver, kidney, and heart as detected by IHC assay (Fig. 8C). The value of CRE of both PBS and exo^scFv/siLPCAT1^ group was less than 10 μmol/L the limits of the reference range, which may due to reduced food intake caused by brain tumors. Above results indicated that high dosage of exo^scFv/siLPCAT1^ did not cause acute toxicity to major organs in nude mice.

**Discussion**

Nanoparticles have been extensively researched as particulate drug carriers, and they have shown excellent results during the past decades. In cancer therapeutics, they offer exciting solutions for improving the efficacy of therapeutic reagents by prolonging their residence time within lesions and simultaneously overcoming side effects by minimizing normal tissue accumulation [[27](#_ENREF_27)]. For example, albumin-bound nanoparticle (nab) paclitaxel exhibits enhanced paclitaxel tissue distribution and tumor penetration, and has become the most successful anti-tumor nanomedicine used in the clinic [[28](#_ENREF_28)]. Nonetheless, for most nanomedicine, the potent toxicity (genotoxicity, immunotoxicity, cellular stress, inflammation) derived from their constituents still significantly limits their translational applications, and novel materials are required. Exosomes have been investigated for their therapeutic potential in pre-clinical studies. Exosomes tend to have different organ tropism based on their cells of origin, which could be utilized for organ-targeted delivery of therapeutic reagents [[29](#_ENREF_29)]. For example, doxorubicin-loaded exosomes further reduced tumor growth with no apparent adverse effects observed with equipotent free drug [[30](#_ENREF_30)]. Moreover, the exosome-based strategy alters drug intracellular trafficking by overcoming drug efflux systems. In a mouse model, exosomes used as carriers to deliver paclitaxel to multiple drug-resistant cancer cells showed increased drug cytotoxicity and potent anticancer effects [[31](#_ENREF_31)]. However, a previous study also suggested that tumor-derived exosomes have tumor-targeting properties only when locally injected into the tumor site [[32](#_ENREF_32)]. To more efficiently deliver their therapeutic payload to target cells, exosomes have been modified using two major approaches: exogenously modifying the surface of exosomes or genetically expressing targeting moieties in the original cells to produce active targeted exosomes. For instance, A33-positive exosomes could form a complex with A33 antibody-coated superparamagnetic iron oxide nanoparticles (SPIONs) and thereby gain targetability towards A33-positive colon cancer cells [[33](#_ENREF_33)]. Upon conjugating with transferrin-modified SPIONs through transferrin-transferrin receptor interaction, TNF-α coated exosomes exhibited a prolonged circulation time and enhanced *in vivo* antitumor activity [[34](#_ENREF_34)]. Exosomes surface can also be manipulated to display targeting peptides or antibodies, which are expressed as fusions with exosomal membrane-associated domains such as lamp2b or C1C2, to achieve tissue targeting. In Alzheimer's disease research, RVG peptide expressed with lamp2b protein on the exosomal surface can introduce target delivery of BACE1 siRNA into mice brain and demonstrate efficient gene silencing [[25](#_ENREF_25)]. Anti-EGFR nanobodies fused with glycosylphosphatidylinositol (GPI)-anchor signal peptide enriched on the surface of exosomes increase the binding of exosomes to EGFR positive tumor cells [[35](#_ENREF_35)]. CAR exosomes, released from EGFR or HER2 scFv-transduced CAR-T cells, induce potent antitumor inhibition in target cancer cell lines and solid tumor xenografts [[36](#_ENREF_36)]. In the present study, we engineered HEK293T cells to express the fusion protein of scFv-lamp2b to produce tumor-targeting enhanced exosomes. This scFv has been demonstrated to efficiently deliver siRNA or SPOINs into EGFR positive lung cancer cells both *in vitro* and *in vivo* [[18](#_ENREF_18), [19](#_ENREF_19)]. Our *in vitro* results showed that the functionalized exo^scFv^ could bind to EGFR positive PC9 cells more efficiently than exo^ctrl^, and exo^scFv/siLPCAT1^ caused a significant loss in cell viability compared to the cells treated with the control.

Brain metastases (BM) occur in up to 30%-50% of patients with advanced NSCLC, especially in patients with EGFR positive mutations, such as 21exon L858R [[37](#_ENREF_37)]. Nevertheless, few targeted TKIs can efficiently pass the BBB, and neither can chemotherapies. Exosomes have the capacity to cross the BBB, yet modifying them with ligands specific to the lesion area rather than the whole brain will accelerate the therapeutic applications of exosomes in brain diseases. Therefore, we next sought to determine whether surface modification of exosomes with specific scFv will affect gene silencing efficiency in the brain. Several kinds of animal models have been used in researching cancer BM, including intraparenchymal implantation [[24](#_ENREF_24)], intracardiac injection [[38](#_ENREF_38)], intracarotid artery injection [[39](#_ENREF_39)], and spontaneous formation [[40](#_ENREF_40)]. Directly intracardiac injection of tumor cells is often associated with undesirable complications such as artery thromboembolism, and spontaneous formation of BM is often associated with lower odds of success. In the other hand, our purpose was not to explore the mechanism of lung cancer BM, but to verify whether the exo^scFv^ hold potential for targeting and suppressing intracranial after crossing blood-brain barrier. Therefore, we chose intraparenchymal implantation to construct the BM model by injecting lung cancer cells directly into the brain parenchyma of mice mounted on a brain stereotaxie apparatus. The results showed that we provided a stable method to display EGFR-specific scFv on the surface of exosomes. Exo^scFv^ exhibited accelerated BBB penetration and prolonged tumor accumulation in BM lesions compared to exo^ctrl^, indicating increased gene silencing efficacy and diminished adverse effects. However, xenograte tumors in brain were great burden to mice, especially to control groups, and resulted in poor performance status, like weight loss and hypoactivity. Thus, we shortened the observation period as much as possible.

The current standard of care for locally advanced or metastatic NSCLC includes targeted therapy, chemotherapy, radiotherapy, and immunotherapy, but drug resistance within the tumor has crippled these treatments. RNAi technology (RNAi), especially siRNA, could be an alternative to control tumor growth by specifically silencing oncogenes [[41](#_ENREF_41)]. However, naked siRNA usually lacks specificity, biocompatibility, and stability in blood circulation [[42](#_ENREF_42)]. To preserve the integrity and functionality, siRNA has been encapsulated in carriers, including various nanoparticles, to avoid degradation from nucleases. In addition, siRNA-loaded nanoparticles have been conjugated with antibody or peptide ligands targeting specific biomarkers in tumors to improve specificity [[43](#_ENREF_43)]. LPCAT1 was upregulated in NSCLC and was associated with poor prognosis of patients, partly by promoting CNS metabolism [[14](#_ENREF_14)]. In our study, a great antitumor effect was observed after treatment of EGFR positive lung cancer cells with exo^scFv/siLPCAT1^ compared to exo^scFv/siNC^ both i*n vivo* and *in vitro*. Importantly, our method is safe to deliver bioactive cargos, and no signs of systemic toxicity or side effects were observed, probably because no chemical modification was involved.

**Conclusion**

In the present study, we demonstrated a method for preparing EGFR-specific scFv-anchored exosomes that showed enhanced anticancer activity. The scFv, which was C-terminally fused to lamp2b via genetic engineering, is an ideal tool for delivering therapeutic and diagnostic reagents to tumor sites. The EPR effect of exosomes and active targeting properties of scFv enable exo^scFv^ to gain favorable targeting properties. The retention time of exosomes in subcutaneous tumors was significantly prolonged by scFv modification. Moreover, our exo^scFv^ successfully transported LPCAT1 siRNA across the BBB to targeted BM sites and demonstrated potent antitumor efficacy with negligible toxicity. Taken together, our results highlight that the exosome-based EGFR targeting siRNA delivering system is a promising therapeutic strategy for BM of lung cancer.

**Acknowledgements**

We would like to thank the researchers contributing to public database (GSE131907) and those providing data related to human medicine. We especially thank Dr. Y.H. Hu (Singleron Biotech. Co., LTD, Nanjing, CHN) for the scRNA data processing and analyzing. This work was funded by Basic Research Program of Jiangsu Province (Natural Science Foundation, No. BK20201484), Key Research and Development Projects of Shaanxi Province (No. 2022SF-245) and National Natural Science Foundation of China (No. 81871880).

**Author contributions**

All authors participated in the discussion of the draft. Yuan Lu, Xinling Ren, Rui Zhang, Angang Yang: Conceptualization, Methodology, Supervision, Writing-review & editing; Jun Jiang: Project administration, Data curation, Writing-original draft; Jie Chu, Lu Zhang: Project administration; Xiao Zhang, Zhuo Wan, Shaojie Liu: Investigation; Chao Xu, Kui Liu, Zhenhua Liu: Software, Validation; Jiawei Wang: Visualization.

**Data availability**

The scRNA data (GSE131907) used in this study derives from public datasets. Details can be found in the Methods section of the manuscript.

**Consent for publication**

All authors are Consent for publication.

**Conflict of Interest**

All authors declare no conflict of interest.

**Declarations**

The animal study received approval by the Animal Care and Use Committees of Air Force Medical University for animal welfare. The study was carried out in compliance with the ARRIVE guidelines.

**References**

[1] R.L. Siegel, K.D. Miller, H.E. Fuchs, A. Jemal, Cancer statistics, 2022, CA Cancer J. Clin. 72(1) (2022) 7-33.<https://doi.org/10.3322/caac.21708>

[2] S.L. Wood, M. Pernemalm, P.A. Crosbie, A.D. Whetton, The role of the tumor-microenvironment in lung cancer-metastasis and its relationship to potential therapeutic targets, Cancer Treat. Rev. 40(4) (2014) 558-66.<https://doi.org/10.1016/j.ctrv.2013.10.001>

[3] J.S. Barnholtz-Sloan, A.E. Sloan, F.G. Davis, F.D. Vigneau, P. Lai, R.E. Sawaya, Incidence proportions of brain metastases in patients diagnosed (1973 to 2001) in the Metropolitan Detroit Cancer Surveillance System, J. Clin. Oncol. 22(14) (2004) 2865-72.<https://doi.org/10.1200/jco.2004.12.149>

[4] A.F. Eichler, E. Chung, D.P. Kodack, J.S. Loeffler, D. Fukumura, R.K. Jain, The biology of brain metastases-translation to new therapies, Nat. Rev. Clin. Oncol. 8(6) (2011) 344-56.<https://doi.org/10.1038/nrclinonc.2011.58>

[5] D.A. Cross, S.E. Ashton, S. Ghiorghiu, C. Eberlein, C.A. Nebhan, P.J. Spitzler, J.P. Orme, M.R. Finlay, R.A. Ward, M.J. Mellor, G. Hughes, A. Rahi, V.N. Jacobs, M. Red Brewer, E. Ichihara, J. Sun, H. Jin, P. Ballard, K. Al-Kadhimi, R. Rowlinson, T. Klinowska, G.H. Richmond, M. Cantarini, D.W. Kim, M.R. Ranson, W. Pao, AZD9291, an irreversible EGFR TKI, overcomes T790M-mediated resistance to EGFR inhibitors in lung cancer, Cancer Discov. 4(9) (2014) 1046-61.<https://doi.org/10.1158/2159-8290.Cd-14-0337>

[6] F. Hsu, A. De Caluwe, D. Anderson, A. Nichol, T. Toriumi, C. Ho, EGFR mutation status on brain metastases from non-small cell lung cancer, Lung Cancer 96 (2016) 101-7.<https://doi.org/10.1016/j.lungcan.2016.04.004>

[7] D. Rangachari, N. Yamaguchi, P.A. VanderLaan, E. Folch, A. Mahadevan, S.R. Floyd, E.J. Uhlmann, E.T. Wong, S.E. Dahlberg, M.S. Huberman, D.B. Costa, Brain metastases in patients with EGFR-mutated or ALK-rearranged non-small-cell lung cancers, Lung Cancer 88(1) (2015) 108-11.<https://doi.org/10.1016/j.lungcan.2015.01.020>

[8] A.K. Biswas, S. Han, Y. Tai, W. Ma, C. Coker, S.A. Quinn, A.R. Shakri, T.J. Zhong, H. Scholze, G.G. Lagos, A. Mela, K. Manova-Todorova, E. de Stanchina, A.A. Ferrando, C. Mendelsohn, P. Canoll, H.A. Yu, P.K. Paik, A. Saqi, C.A. Shu, M.G. Kris, J. Massague, S. Acharyya, Targeting S100A9-ALDH1A1-Retinoic Acid Signaling to Suppress Brain Relapse in EGFR-Mutant Lung Cancer, Cancer Discov. 12(4) (2022) 1002-1021.<https://doi.org/10.1158/2159-8290.Cd-21-0910>

[9] J. Fares, I. Ulasov, P. Timashev, M.S. Lesniak, Emerging principles of brain immunology and immune checkpoint blockade in brain metastases, Brain 144(4) (2021) 1046-1066.<https://doi.org/10.1093/brain/awab012>

[10] L. Mashouri, H. Yousefi, A.R. Aref, A.M. Ahadi, F. Molaei, S.K. Alahari, Exosomes: composition, biogenesis, and mechanisms in cancer metastasis and drug resistance, Mol. Cancer 18(1) (2019) 75.<https://doi.org/10.1186/s12943-019-0991-5>

[11] Q. Zhan, K. Yi, X. Cui, X. Li, S. Yang, Q. Wang, C. Fang, Y. Tan, L. Li, C. Xu, X. Yuan, C. Kang, Blood exosomes-based targeted delivery of cPLA2 siRNA and metformin to modulate glioblastoma energy metabolism for tailoring personalized therapy, Neuro Oncol. 24(11) (2022) 1871-1883.<https://doi.org/10.1093/neuonc/noac071>

[12] M. Liu, Y. Hu, G. Chen, The Antitumor Effect of Gene-Engineered Exosomes in the Treatment of Brain Metastasis of Breast Cancer, Front. Oncol. 10 (2020) 1453.<https://doi.org/10.3389/fonc.2020.01453>

[13] J. Shao, J. Zaro, Y. Shen, Advances in Exosome-Based Drug Delivery and Tumor Targeting: From Tissue Distribution to Intracellular Fate, International journal of nanomedicine 15 (2020) 9355-9371.<https://doi.org/10.2147/ijn.S281890>

[14] C. Wei, X. Dong, H. Lu, F. Tong, L. Chen, R. Zhang, J. Dong, Y. Hu, G. Wu, X. Dong, LPCAT1 promotes brain metastasis of lung adenocarcinoma by up-regulating PI3K/AKT/MYC pathway, J. Exp. Clin. Cancer Res. 38(1) (2019) 95.<https://doi.org/10.1186/s13046-019-1092-4>

[15] J. Bi, T.A. Ichu, C. Zanca, H. Yang, W. Zhang, Y. Gu, S. Chowdhry, A. Reed, S. Ikegami, K.M. Turner, W. Zhang, G.R. Villa, S. Wu, O. Quehenberger, W.H. Yong, H.I. Kornblum, J.N. Rich, T.F. Cloughesy, W.K. Cavenee, F.B. Furnari, B.F. Cravatt, P.S. Mischel, Oncogene Amplification in Growth Factor Signaling Pathways Renders Cancers Dependent on Membrane Lipid Remodeling, Cell metabolism 30(3) (2019) 525-538.e8.<https://doi.org/10.1016/j.cmet.2019.06.014>

[16] T. Zhao, Y. Zhang, X. Ma, L. Wei, Y. Hou, R. Sun, J. Jiang, Elevated expression of LPCAT1 predicts a poor prognosis and is correlated with the tumour microenvironment in endometrial cancer, Cancer Cell Int. 21(1) (2021) 269.<https://doi.org/10.1186/s12935-021-01965-1>

[17] M. Tao, J. Luo, T. Gu, X. Yu, Z. Song, Y. Jun, H. Gu, K. Han, X. Huang, W. Yu, S. Sun, Z. Zhang, L. Liu, X. Chen, L. Zhang, C. Luo, Q. Wang, LPCAT1 reprogramming cholesterol metabolism promotes the progression of esophageal squamous cell carcinoma, Cell Death Dis. 12(9) (2021) 845.<https://doi.org/10.1038/s41419-021-04132-6>

[18] Y. Lu, L. Liu, Y. Wang, F. Li, J. Zhang, M. Ye, H. Zhao, X. Zhang, M. Zhang, J. Zhao, B. Yan, A. Yang, H. Feng, R. Zhang, X. Ren, siRNA delivered by EGFR-specific scFv sensitizes EGFR-TKI-resistant human lung cancer cells, Biomaterials 76 (2016) 196-207.<https://doi.org/10.1016/j.biomaterials.2015.10.036>

[19] Y. Lu, J. Huang, F. Li, Y. Wang, M. Ding, J. Zhang, H. Yin, R. Zhang, X. Ren, EGFR-specific single-chain variable fragment antibody-conjugated Fe(3)O(4)/Au nanoparticles as an active MRI contrast agent for NSCLC, MAGMA 34(4) (2021) 581-591.<https://doi.org/10.1007/s10334-021-00916-1>

[20] N. Kim, H.K. Kim, K. Lee, Y. Hong, J.H. Cho, J.W. Choi, J.I. Lee, Y.L. Suh, B.M. Ku, H.H. Eum, S. Choi, Y.L. Choi, J.G. Joung, W.Y. Park, H.A. Jung, J.M. Sun, S.H. Lee, J.S. Ahn, K. Park, M.J. Ahn, H.O. Lee, Single-cell RNA sequencing demonstrates the molecular and cellular reprogramming of metastatic lung adenocarcinoma, Nature communications 11(1) (2020) 2285.<https://doi.org/10.1038/s41467-020-16164-1>

[21] Y. Zhang, B. Li, J. Duan, X. Chen, X. Zhang, J. Ye, A. Veloso, J. Fan, N.J.b. Fang, SynEcoSys: a multifunctional platform of large-scale single-cell omics data analysis, (2023) 2023.02. 14.528566

[22] B. You, W. Xu, B. Zhang, Engineering exosomes: a new direction for anticancer treatment, American journal of cancer research 8(8) (2018) 1332-1342

[23] J. Jiang, Y. Lu, F. Zhang, T. Pan, Z. Zhang, Y. Wan, X. Ren, R. Zhang, Semaphorin 4B promotes tumor progression and associates with immune infiltrates in lung adenocarcinoma, BMC Cancer 22(1) (2022) 632.<https://doi.org/10.1186/s12885-022-09696-w>

[24] O. Sampetrean, I. Saga, M. Nakanishi, E. Sugihara, R. Fukaya, N. Onishi, S. Osuka, M. Akahata, K. Kai, H. Sugimoto, A. Hirao, H. Saya, Invasion precedes tumor mass formation in a malignant brain tumor model of genetically modified neural stem cells, Neoplasia (New York, N.Y.) 13(9) (2011) 784-91.<https://doi.org/10.1593/neo.11624>

[25] L. Alvarez-Erviti, Y. Seow, H. Yin, C. Betts, S. Lakhal, M.J. Wood, Delivery of siRNA to the mouse brain by systemic injection of targeted exosomes, Nat. Biotechnol. 29(4) (2011) 341-5.<https://doi.org/10.1038/nbt.1807>

[26] H. Broder, A. Anderson, T.J. Kremen, S.K. Odesa, L.M. Liau, MART-1 adenovirus-transduced dendritic cell immunization in a murine model of metastatic central nervous system tumor, J. Neurooncol. 64(1-2) (2003) 21-30.<https://doi.org/10.1007/bf02700017>

[27] G. Wei, Y. Wang, G. Yang, Y. Wang, R. Ju, Recent progress in nanomedicine for enhanced cancer chemotherapy, Theranostics 11(13) (2021) 6370-6392.<https://doi.org/10.7150/thno.57828>

[28] N. Chen, C. Brachmann, X. Liu, D.W. Pierce, J. Dey, W.S. Kerwin, Y. Li, S. Zhou, S. Hou, M. Carleton, R.A. Klinghoffer, M. Palmisano, R. Chopra, Albumin-bound nanoparticle (nab) paclitaxel exhibits enhanced paclitaxel tissue distribution and tumor penetration, Cancer Chemother. Pharmacol. 76(4) (2015) 699-712.<https://doi.org/10.1007/s00280-015-2833-5>

[29] O.P. Wiklander, J.Z. Nordin, A. O'Loughlin, Y. Gustafsson, G. Corso, I. Mäger, P. Vader, Y. Lee, H. Sork, Y. Seow, N. Heldring, L. Alvarez-Erviti, C.I. Smith, K. Le Blanc, P. Macchiarini, P. Jungebluth, M.J. Wood, S.E. Andaloussi, Extracellular vesicle in vivo biodistribution is determined by cell source, route of administration and targeting, Journal of extracellular vesicles 4 (2015) 26316.<https://doi.org/10.3402/jev.v4.26316>

[30] S.C. Jang, O.Y. Kim, C.M. Yoon, D.S. Choi, T.Y. Roh, J. Park, J. Nilsson, J. Lötvall, Y.K. Kim, Y.S. Gho, Bioinspired exosome-mimetic nanovesicles for targeted delivery of chemotherapeutics to malignant tumors, ACS nano 7(9) (2013) 7698-710.<https://doi.org/10.1021/nn402232g>

[31] M.S. Kim, M.J. Haney, Y. Zhao, V. Mahajan, I. Deygen, N.L. Klyachko, E. Inskoe, A. Piroyan, M. Sokolsky, O. Okolie, S.D. Hingtgen, A.V. Kabanov, E.V. Batrakova, Development of exosome-encapsulated paclitaxel to overcome MDR in cancer cells, Nanomedicine 12(3) (2016) 655-664.<https://doi.org/10.1016/j.nano.2015.10.012>

[32] M. Morishita, Y. Takahashi, M. Nishikawa, Y. Takakura, Pharmacokinetics of Exosomes-An Important Factor for Elucidating the Biological Roles of Exosomes and for the Development of Exosome-Based Therapeutics, J. Pharm. Sci. 106(9) (2017) 2265-2269.<https://doi.org/10.1016/j.xphs.2017.02.030>

[33] Y. Li, Y. Gao, C. Gong, Z. Wang, Q. Xia, F. Gu, C. Hu, L. Zhang, H. Guo, S. Gao, A33 antibody-functionalized exosomes for targeted delivery of doxorubicin against colorectal cancer, Nanomedicine 14(7) (2018) 1973-1985.<https://doi.org/10.1016/j.nano.2018.05.020>

[34] M. Zhuang, X. Chen, D. Du, J. Shi, M. Deng, Q. Long, X. Yin, Y. Wang, L. Rao, SPION decorated exosome delivery of TNF-α to cancer cell membranes through magnetism, Nanoscale 12(1) (2020) 173-188.<https://doi.org/10.1039/c9nr05865f>

[35] S.A. Kooijmans, C.G. Aleza, S.R. Roffler, W.W. van Solinge, P. Vader, R.M. Schiffelers, Display of GPI-anchored anti-EGFR nanobodies on extracellular vesicles promotes tumour cell targeting, Journal of extracellular vesicles 5 (2016) 31053.<https://doi.org/10.3402/jev.v5.31053>

[36] W. Fu, C. Lei, S. Liu, Y. Cui, C. Wang, K. Qian, T. Li, Y. Shen, X. Fan, F. Lin, M. Ding, M. Pan, X. Ye, Y. Yang, S. Hu, CAR exosomes derived from effector CAR-T cells have potent antitumour effects and low toxicity, Nature communications 10(1) (2019) 4355.<https://doi.org/10.1038/s41467-019-12321-3>

[37] A. Boire, P.K. Brastianos, L. Garzia, M. Valiente, Brain metastasis, Nat. Rev. Cancer 20(1) (2020) 4-11.<https://doi.org/10.1038/s41568-019-0220-y>

[38] Y. Kienast, L. von Baumgarten, M. Fuhrmann, W.E. Klinkert, R. Goldbrunner, J. Herms, F. Winkler, Real-time imaging reveals the single steps of brain metastasis formation, Nat. Med. 16(1) (2010) 116-22.<https://doi.org/10.1038/nm.2072>

[39] I.J. Fidler, G. Schackert, R.D. Zhang, R. Radinsky, T. Fujimaki, The biology of melanoma brain metastasis, Cancer Metastasis Rev. 18(3) (1999) 387-400.<https://doi.org/10.1023/a:1006329410433>

[40] W. Cruz-Munoz, S. Man, P. Xu, R.S. Kerbel, Development of a preclinical model of spontaneous human melanoma central nervous system metastasis, Cancer Res. 68(12) (2008) 4500-5.<https://doi.org/10.1158/0008-5472.Can-08-0041>

[41] X. Chen, L.S. Mangala, C. Rodriguez-Aguayo, X. Kong, G. Lopez-Berestein, A.K. Sood, RNA interference-based therapy and its delivery systems, Cancer Metastasis Rev. 37(1) (2018) 107-124.<https://doi.org/10.1007/s10555-017-9717-6>

[42] K.A. Whitehead, R. Langer, D.G. Anderson, Knocking down barriers: advances in siRNA delivery, Nature reviews. Drug discovery 8(2) (2009) 129-38.<https://doi.org/10.1038/nrd2742>

[43] S. El-Andaloussi, Y. Lee, S. Lakhal-Littleton, J. Li, Y. Seow, C. Gardiner, L. Alvarez-Erviti, I.L. Sargent, M.J. Wood, Exosome-mediated delivery of siRNA in vitro and in vivo, Nat. Protoc. 7(12) (2012) 2112-26.<https://doi.org/10.1038/nprot.2012.131>
